# Significant roles in RNA-binding for the amino-terminal regions of Drosophila Pumilio and Nanos

**DOI:** 10.1371/journal.pgen.1011616

**Published:** 2025-03-31

**Authors:** Tammy H. Wharton, Mohammad Marhabaie, Robin P. Wharton

**Affiliations:** Department of Molecular Genetics, Department of Cancer Biology and Genetics, Center for RNA Biology, Ohio State University, Columbus, Ohio, United States of America; a Current address: The Steve and Cindy Rasmussen Institute for Genomic Medicine, Abigail Wexner Research Institute at Nationwide Children’s Hospital, Columbus, Ohio, United States of America; Princeton University, UNITED STATES OF AMERICA

## Abstract

The Drosophila Pumilio (Pum) and Nanos (Nos) RNA-binding proteins govern abdominal segmentation in the early embryo, as well as a variety of other events during development. They bind together to a compound Nanos Response Element (NRE) present in thousands of maternal mRNAs in the ovary and embryo, including *hunchback* (*hb*) mRNA, thereby regulating poly-adenylation, translation, and stability. Many studies support a model in which mRNA recognition and effector recruitment are carried out by distinct regions of each protein. The well-ordered Pum and Nos RNA-binding domains (RBDs) are sufficient to specifically recognize NREs; the larger intrinsically disordered N-terminal regions (NTRs) of each protein have been thought to act by recruiting mRNA regulators. Here we use yeast interaction assays and experiments testing the regulation of *hb* mRNA in vivo to show that the NTRs play a significant role in recognition of the NRE, acting via two mechanisms. First, the Pum and Nos NTRs interact in trans to promote assembly of the Pum/Nos/NRE ternary complex. Second, the Pum NTR acts via an unknown mechanism in cis, modifying NRE recognition by its RBD. The ability of the NTR to alter binding to the NRE is conserved in human Pum2.

## Author summary

Many of the early developmental decisions in the fruit fly embryo are controlled by mRNAs made by the female and deposited in the egg before fertilization. Some of these mRNAs are differentially regulated, spatially or temporally, as part of the program that drives patterning of the embryo. Here, we study two of the proteins that target maternal mRNAs for regulation-- Pumilio and Nanos. These factors collaborate to bind jointly to particular sequences in target mRNAs. Pumilio and Nanos each have two domains: a compact, well-ordered domain that recognizes mRNA targets, and a large low-complexity domain made up of only a few of the twenty amino acids that is thought to be amorphous. In this report, we show that the amorphous domains of Pumilio and Nanos in fact play important roles in the recognition of mRNA targets, acting at two different levels. At one level, the amorphous domains of Pumilio and Nanos interact with each other, stabilizing joint binding to mRNA targets. At another level, the amorphous domain of Pumilio modifies the properties of its RNA-contacting domain, changing the way it interacts with sequences in the RNA via a currently unknown mechanism.

## Introduction

Translational regulation by Pum and Nos plays an important role in Drosophila, particularly at three stages in development of the ovarian germline and the early embryo. First, the initial step in formation of an egg chamber involves the division of a germline stem cell; maintenance of stem cell status requires Pum and Nos activity [1,2]. Second, from late in oogenesis to the onset of zygotic transcription in the embryo, Pum and Nos jointly regulate thousands of mRNAs to sculpt the maternal transcriptome [3]. Third, Pum and Nos jointly regulate maternal *hb* mRNA in the posterior of the syncytial cleavage stage embryo to allow abdominal segmentation [4,5]. In addition to these three functions, Pum and Nos govern many other biological processes in Drosophila, including various aspects of the cell biology of primordial germ cells (PGCs) [6–8], dendritic arborization of larval sensory neurons [9,10], and remodeling of the larval neuromuscular junction [11].

**Fig 1:**
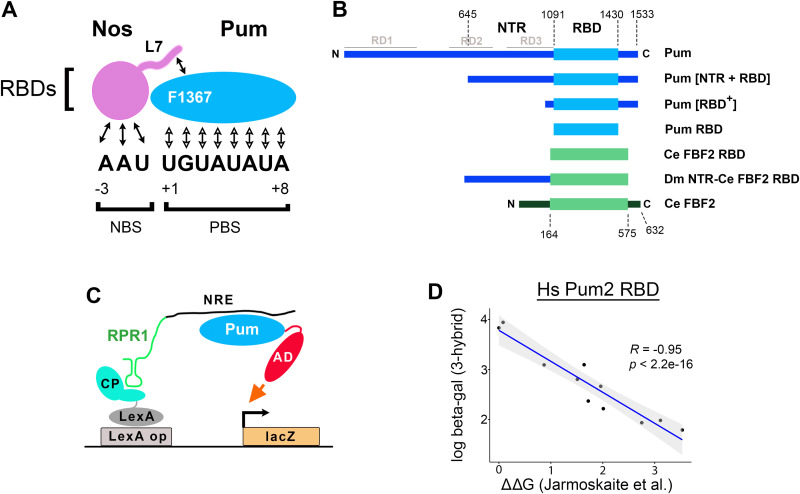
The yeast three-hybrid assay of RNA-binding. A. Schematic drawing of the Nos and Pum RBDs bound to the wild type NRE used in this and subsequent figures. The drawing is based on the structure of Weidmann and colleagues [5], and emphasizes interactions that stabilize the ternary complex between the C-terminal tail of Nos (which is altered in the L7 mutant) and F1367 in Pum, as well as interactions of Nos with the RNA. Interactions between Pum and the RNA are shown with open arrowheads. B. A drawing to scale of proteins tested in this work, with amino acid residue landmarks above and names to the right. Full-length wt Pum corresponds to the PA protein isoform; boundaries of its NTR (as defined functionally in this work) and minimal RBD are indicated above. RD1, RD2, and RD3 indicate repression domains in Pum that can mediate regulation when tethered to reporters via an exogenous RBD [17]. C. A schematic drawing of the yeast three-hybrid assay that measures RNA-binding, as originally described by Sengupta and colleagues [18]. D. Correlation between binding of the Hs Pum2 RBD to a wt NRE and 10 different mutant sites, measured in the yeast three-hybrid experiments described later in this report (log units of β-galactosidase on the y-axis) and in vitro by Jarmoskaite and colleagues (free energy of binding relative to binding to the wt NRE) [19]. Light shading indicates 95% confidence limits. Underlying data are in S1 Data.

Although Nos and Pum may regulate some mRNAs independently of each other, in most cases they act together, binding jointly to a compound binding site in target mRNAs [3,12]. This compound site, a Nanos Response Element (NRE), is comprised of adjacent Nos and Pum binding sites (NBS and PBS, respectively; see Fig 1A) [4,5]. The Pum RBD, which resides in the C-terminal portion of the protein, consists of 8 homologous repeats that collectively constitute a Puf domain. Pum binds on its own with high specificity and affinity to the PBS [13,14], with each of the Puf repeats recognizing one of the 8 nucleotides in the PBS [15]. Unlike Pum, the Nos RBD on its own does not bind to the NBS [4,5,16]. But together, the two RBDs bind cooperatively to the NRE, with Nos contributing modest sequence specificity via contacts to the degenerate NBS as well as binding energy that stabilizes the ternary Nos/Pum/NRE complex. As a result of protein-protein interactions between the Nos and Pum RBDs, the specificity of Pum for nucleotides at the 3’-end of the PBS (positions 6-8) is relaxed in the ternary complex, and this is thought to allow regulation of an expanded repertoire of mRNAs [5].

Both Pum and Nos are bipartite proteins, with carboxy-proximal RBDs fused to larger N-terminal regions (NTRs) that are involved in the recruitment of effectors that regulate translation and stability of bound mRNAs. The major effector recruited by Nos+Pum is thought to be the CCR4/Not/deadenylase complex (henceforth, the deadenylase complex) [17,20,21]. The full-length, 1091 residue Pum NTR contains three repression domains (RD1 - RD3 in Fig 1B) that can regulate bound mRNAs when tethered to a suitably engineered reporter in transfected cells; each RD also interacts with the deadenylase complex in a number of different experiments [17,22]. The NTR of Nos also interacts with the deadenylase complex and represses reporter mRNAs when tethered via an exogenous RNA-binding protein [21]. The Pum RBD may provide auxiliary contacts that help recruit the deadenylase complex [23]. However, the general picture to emerge from the work summarized above is that, the Pum and Nos RBDs are responsible for mRNA target recognition, while the Pum and Nos NTRs interact with the deadenylase complex that is responsible for regulation of mRNA translation and stability.

Studies of the function of the Pum and Nos NTRs have primarily been performed in cultured cells or in embryos [13,16,17,21]. The Pum and Nos NTRs are comprised of relatively large, intrinsically disordered regions (IDRs), which has hindered studies in vitro. In contrast, the Pum and Nos RBDs are compact, folded domains, permitting the extensive molecular and structural experiments that have led to our current understanding of their role in RNA sequence recognition [5]. To our knowledge, no role in RNA recognition has been ascribed previously to the Nos and Pum NTRs.

In this report, we use yeast interaction experiments to investigate function of the Nos and Pum NTRs in RNA-binding. We find that, in addition to their previously described roles in effector recruitment, the NTRs have two important functions in target recognition. First, intermolecular interactions between the Nos and Pum NTRs contribute to RNA-binding and site selection. Second, we find a surprising intramolecular effect of the Pum NTR on the apparent affinity and binding specificity of its associated RBD. We show that the intramolecular activity of the NTR plays a role in regulation of *hb* mRNA in the Drosophila embryo.

## Results

As described above, Nos and Pum bind jointly to a compound 11 nt RNA sequence consisting of adjacent binding sites for each protein (Fig 1A). In addition to interactions made by each RBD to the RNA, formation of the ternary complex is dependent on interactions between the two RBDs, involving F1367 in Pum and residues in the C-terminal tail of Nos that are deleted in the L7 mutant (Fig 1A) [4,5,24]. Based on studies with the isolated RBDs, the identities of RNA bases at positions +1 through +4, UGUA, have been thought to be rigidly specified, such that substitutions at these nucleotides significantly reduce binding of either Pum alone or joint binding of Nos+Pum. In particular, substitution of U for A at position +4 (henceforth, a 4U NRE) lowers the affinity of the homologous Homo sapiens Pum2 (Hs Pum2) RBD approximately tenfold [19].

We were therefore surprised to find that Nos+Pum jointly target mRNAs in the ovary and embryo via “mutant” 4U NREs, as well as via wildtype NREs [3]. One possible explanation for the apparent discrepancy between the in vitro and in vivo experiments outlined above is that the Drosophila and human Pum RBDs have substantially different binding specificities. Another possibility is that residues outside the Nos and Pum RBDs alter RNA site selection, since in the experiments of Marhabaie and colleagues [3], regulation is under the control of the endogenous, full-length proteins. The RBD constitutes only 22% of the main Pum isoform in the early embryo protein (Fig 1B), and the contribution of the remaining 78% of the residues to binding has not been clear.

### Yeast three-hybrid experiments to measure Pum binding to RNA

To test the possibilities described above, we first wished to compare the binding of Pum with and without its NTR to the wildtype NRE and various mutant derivatives, including the 4U site. To date, we have been unable to prepare soluble Pum (or Nos) derivatives bearing appreciable portions of their respective NTRs to measure binding in vitro. However, we have successfully expressed longer portions of Pum and full-length Nos in yeast (as described below). We therefore turned to yeast three-hybrid experiments (Fig 1C) [18] to measure binding of Pum to various NREs. In these experiments, Pum binding to the NRE recruits a tethered transcriptional activation domain (AD) to the promoter of a β-galactosidase reporter gene. A key component of the experiment is a fragment of RPR1, a non-protein coding RNA that is transcribed by Pol III and retained in the nucleus.

Three hybrid experiments have been used extensively to measure the RNA binding of Puf domain proteins including Pum, C. elegans FBF2, and Hs Pum1 [24–27]. We further validated the approach by measuring β-galactosidase levels driven by binding of the Hs Pum2 RBD to a panel of singly mutant NREs (in experiments described below) and then compared our results with those from a high-throughput, thermodynamically rigorous measurement of binding in vitro from the Herschlag lab [19]. As shown in Fig 1D, for a panel of 11 NREs, there is an excellent correlation (R = -0.95) between our results and the relative affinities measured in vitro by Jarmoskaite and colleagues.

### The Pum NTR alters RNA-binding

**Fig 2:**
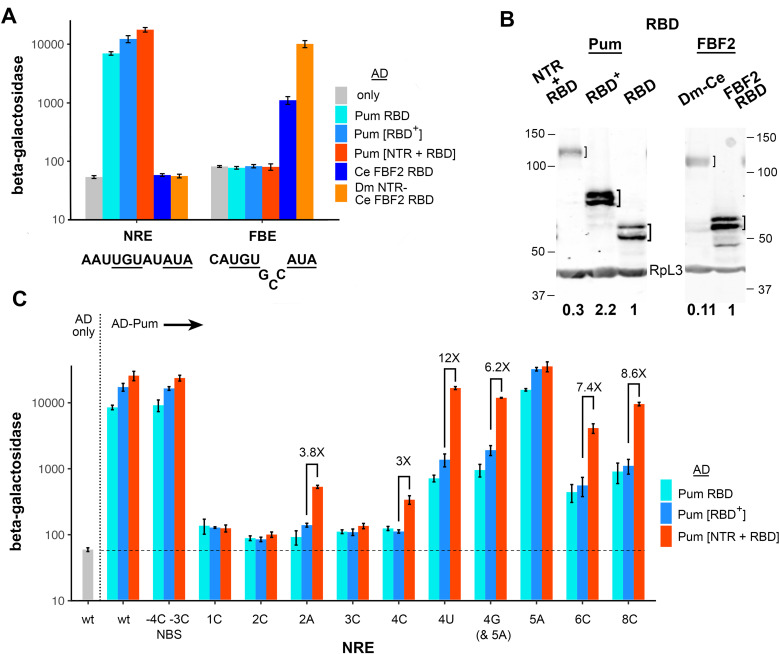
The Pum NTR alters recognition of RNA. A. Three-hybrid assays measuring the binding of the 5 Puf domain proteins indicated to the right to either the wt Drosophila NRE or one of the FBE sites in *gld*-1 mRNA. For all fusions in this figure, transcriptional activation is dependent on the vector-encoded GAL4 AD (see S2 Data for these and other controls). Nucleotides conserved in the two sites are underlined. The bases at positions +4 through +6 in the FBE loop out on binding to FBF2 and do not contact the protein [25]. Note that the C at position -2 of the FBE is required for optimal binding. Here and in subsequent figures, the y-axis is units of β-galactosidase in arbitrary light units (X 1000); the scale is logarithmic; and each measurement is the average of 3 or 4 independent cultures, with error bars showing standard deviation. B. Western blots showing the relative level (displayed in bold below) of each protein assayed in A. Proteins are detected via a shared, vector-encoded HA epitope. Quantitation is derived from measurements of 3 or 4 samples on a single blot here and in subsequent figures, with protein loading in each lane normalized to the level of RpL3. Note that phosphorylation of the GAL4 AD at Serine 837 generates a low mobility isoform that is well-resolved from the unmodified AD for most low MW fusions [29,30]. The corresponding DBD fusions run as single bands. Here and throughout the rest of this work, the labelled hash marks beside each Western blot show the mobility of molecular weight markers in kDa. C. Binding of the three AD-Pum fusions indicated at the right to various derivatives of a *hb* NRE in yeast three-hybrid experiments. Selected comparisons between β-galactosidase levels (based on the values in S2 Data) in the presence or absence of the NTR are highlighted with brackets, with the fold-difference indicated above each bracket (i.e., “3X”). The x-axis displays the RNA site in each group of experiments here and in other figures that show the results of three- or four-hybrid RNA-binding experiments. Sequence of the wild type (wt) site is in Fig 1A; the NBS mutant has A to C substitutions at two positions, -4 and -3, and has been assayed for activity in vivo [13]. The remaining mutant sites are identified by position within the NRE and identity of the single nucleotide change (e.g., 1C = U to C substitution at position +1). Note that the 4G site also bears a 5A substitution to avoid creation of a fortuitous UGU sequence that would constitute the core of a cryptic Pum binding site. In the empty vector “AD only” control at the left, yeast contained the wt RNA. We have also measured the background level of β-galactosidase in yeast co-expressing AD only with each of the RNAs. In no case is the background level substantially different than with wt RNA. The horizontal dashed line indicates the basal level of β-galactosidase here and in subsequent figures. Underlying data are in S2 Data.

Expression of either full-length Pum or a derivative bearing the 643 most N-terminal residues was toxic or semi-toxic in yeast. But we found that expression of a truncated derivative lacking these residues, Pum[NTR + RBD] (Fig 1B) had little effect on yeast viability or growth. For simplicity, throughout the remainder of this report, we refer to Pum residues 645-1091 as the Pum NTR. For three Pum fragments, we then (1) compared RNA-binding activity and (2) measured accumulation in yeast: the minimal Pum RBD; a slightly larger fragment named Pum[RBD^+^] that is similar to one previously shown to bind specifically in yeast three-hybrid experiments and to partially regulate *hb* in embryos [4]; and Pum [NTR + RBD] (Fig 1B). Expression of each protein was measured in Western blots, detecting a common epitope-tag inserted between the AD and Pum moieties. To assay binding specificity, we measured binding to the Drosophila NRE and, as a control, binding to a related sequence recognized by the C. elegans FBF2 (Fem3 binding factor 2) protein-- the 9 nt FBF2 binding element (FBE) shown in Fig 2A. Previous work has shown that the Pum and FBF2 RBDs bind only to their cognate sites [25,28].

As shown in Fig 2A, all three Pum fragments tested bind to the NRE, stimulating β-galactosidase more than 100-fold above the background level in yeast transformed with the AD empty vector control. In contrast, none of the Pum fragments binds significantly above background to the C. elegans FBE. Thus, the Pum NTR does not detectably promote non-specific binding either to the FBE or to the remainder of the chimeric RNA encoding a portion of the yeast RPR1 RNA in which it is embedded (Fig 1C). As an additional control, we assayed the binding and accumulation of the minimal C. elegans FBF2 RBD and a chimera, in which the Drosophila Pum NTR is fused to the FBF2 RBD (Fig 1B). Note that these proteins lack a recently described auto-inhibitory element found C-terminal to the RBD [31]. As shown in Fig 2A, the FBF2 RBD binds detectably only to its cognate site, the FBE, whether or not it is fused to the Pum NTR. Further control experiments are described in S1 Text.

Although the Pum NTR does not appear to promote non-specific RNA-binding, it does have an effect on the apparent affinity of the RBD to which it is fused in cis. In the case of NRE binding, the NTR stimulates binding 1.4-fold, even though Pum[NTR + RBD] accumulates to approximately 7-fold lower levels in yeast than does Pum[RBD^+^] (Fig 2A and 2B). In the case of FBE binding, the effect of the NTR is even more pronounced: the Dm NTR-Ce FBF2 RBD (henceforth, Dm-Ce) chimera binds 9.2X better than does the isolated FBF2 RBD, even though the chimera accumulates to 9-fold lower levels (Fig 2A and 2B).

We next wished to test the effect of the Pum NTR on binding specificity for various mutant NREs. To this end, we tested the binding of the three Pum fragments assayed in Fig 2A to a panel of mutant sites, each bearing a single substitution at positions 1 through 8 of the Pum binding site (PBS). As a control, we also tested binding to an NRE bearing two mutations in the Nos binding site (NBS). Binding of the minimal Pum RBD agrees with the results of previous experiments that assayed binding in vitro [5] or in ovarian extracts [32]. As shown in Fig 2C, substitutions in the core UGU sequence from +1 to +3 abolish binding, whereas substitutions at more 3’-proximal positions are tolerated (to varying extents) such that specificity at positions +4 to +8 of the NRE is partially relaxed. The binding specificities of Pum[RBD^+^] and the Pum RBD are essentially the same; for none of the tested sites is the difference in binding between these two proteins greater than 2-fold, likely reflecting the 2.2-fold higher level of Pum[RBD^+^] (Fig 2B).

In contrast, the NTR substantially alters binding mediated by the RBD in cis. The apparent binding specificity of Pum[NTR + RBD] is further relaxed at positions +4, +6, and +8, beyond the level seen with the RBD alone, such that most mutations in this portion of the NRE are well tolerated (Fig 2C). The extent to which the NTR stimulates binding to mutant sites is indicated in Fig 2C; in particular, it stimulates binding to the 4U site that mediates Nos+Pum activity in vivo [3] 12-fold, such that Pum[NTR + RBD] binds only 1.5-fold less to the 4U site than to the wild type NRE. As noted above, inclusion of the NTR elevates binding to the wildtype NRE (and the NBS mutant NRE), even though Pum[NTR + RBD] is expressed at a lower level than Pum[RBD^+^]. Taken together, the evidence presented in Fig 2 supports the idea that the Pum NTR alters the binding specificity of the RBD in cis.

### Yeast four-hybrid experiments to measure Nos recruitment to the NRE

**Fig 3:**
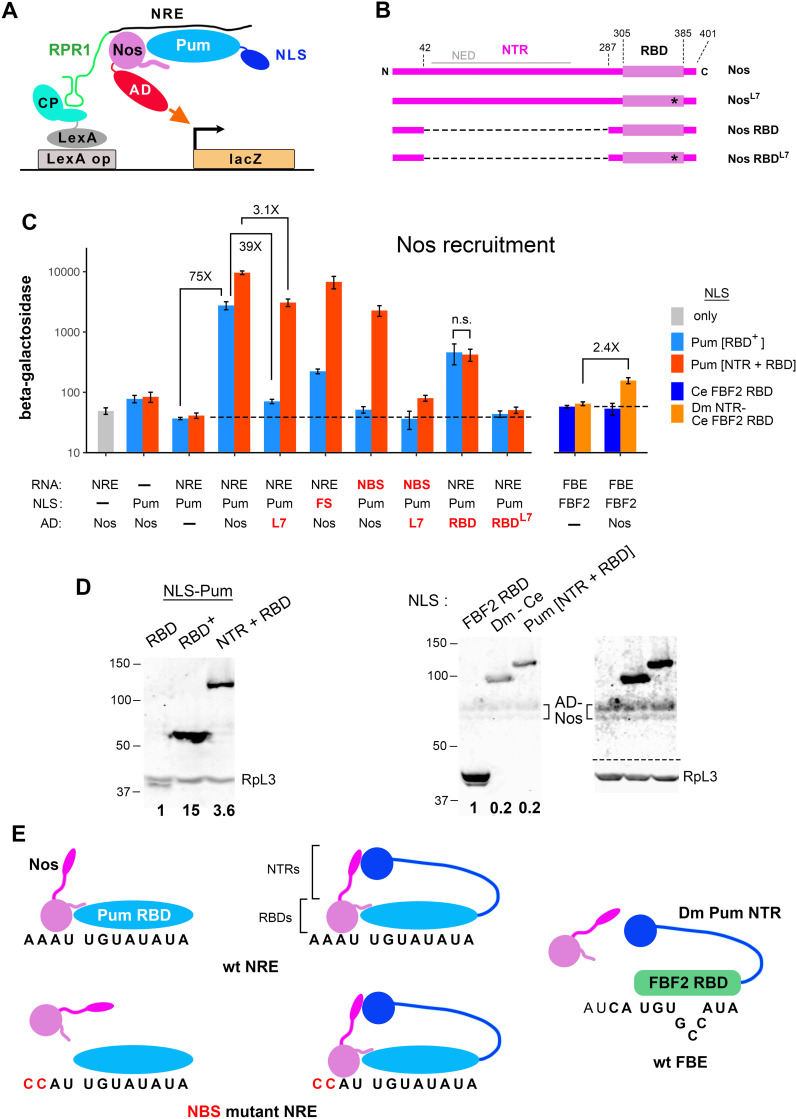
The Pum NTR stimulates recruitment of full-length Nos. A. Schematic drawing of a four-hybrid experiment that measures recruitment of an AD-Nos fusion into a ternary complex with Pum and the NRE. Note that Pum is localized to the nucleus via the SV40 nuclear localization signal (NLS), but the protein has no functional AD. B. Schematic drawing to scale of Nos derivatives used in these experiments. The Nos NTR, as defined functionally, consists of residues 43-286; previous work showed that efficient expression of Nos derivatives in yeast requires the N-terminal 42 residues, which are therefore common to all Nos fragments [4]. The Nos Effector Domain (NED), which mediates interaction with the deadenylase complex [21] is indicated above in grey. The L7 mutation, which is an in-frame deletion of 7 amino acids, is indicated with an asterisk. C. Yeast four-hybrid assays of Nos recruitment. The first three columns are controls showing that Nos does not bind to the NRE detectably in the absence of Pum (column 1); that in the presence of Pum but the absence of the NRE, Nos does not bind detectably to the vector-encoded RPR1 moiety that is part of the chimeric RNA used elsewhere in this experiment (column 2); and that the Pum-bound NRE bait does not autoactivate transcription of the LacZ reporter (column 3). The remaining columns show LacZ reporter transcription due to the recruitment of various AD-Nos fusions; below each column is shown the identity of the three plasmid-encoded components of the four-hybrid experiment, with mutant derivatives in red. The NBS mutant is -4C & -3C, as in Fig 2C, the FBE is as shown in Fig 2A, FS indicates the F1367S substitution in the Pum RBD, and L7 is the deletion in the Nos tail described above. We note that the RNA-binding activities of wt and F1367S mutant Pum are indistinguishable, for both the RBD and [NTR + RBD] protein fragments (S3 Data). The NLS-fusions to various Puf proteins are indicated to the right. Additional controls are in S3 Data. D. Western blots showing the relative level (in bold below) of proteins assayed in C. The blot in the middle shows only signal from detection of the HA epitope tag, since the FBF2 RBD and yeast RpL3 proteins comigrate. At the far right is a composite image of the same blot shown in the middle. Above the dashed line is an over-exposed display of the signal from the HA epitope tag to allow visualization of the low level of AD-Nos present in these experiments. The level of Nos is near the limit of detection and cannot be reliably measured. Over-expression of Nos to a higher level in similar experiments is reported in S1 Fig. Below the dashed line is displayed the signal from detection of RpL3 only. E. One model for how the Nos and Pum NTRs could potentiate binding to the NRE, as described in the text. Although shown as a direct interaction between the NTRs, our experiments do not rule out the possibility that bridging yeast proteins mediate an indirect interaction. Underlying data are in S3 Data.

We next asked whether the NTRs of Pum and Nos influence joint binding of both proteins to form a ternary complex with the NRE. To address this question, we used a four-hybrid variant of the yeast assay described above, in which expression from the LacZ reporter is measured in cells expressing: (1) a chimeric RNA encoding an NRE, (2) Pum derivatives fused to a nuclear localization signal (NLS) but not an AD, and (3) AD-fusions to Nos (Fig 3A). In these experiments, β-galactosidase levels report recruitment of Nos into a ternary complex with Pum and the NRE. We have previously used a similar approach to show recruitment of Nos by Pum to the NRE and by Bruno to RNA containing Bruno binding sites [3,4].

As shown in Fig 3C, Pum[RBD^+^] recruits Nos into a ternary complex with the wild type NRE, stimulating reporter transcription 75-fold above the background level seen in yeast expressing the bait (Pum bound to the NRE) but with an AD-only empty vector control. Nos recruitment is dependent on expression of the Pum RBD and the NRE; thus, even though the Nos RBD has a high non-specific affinity for RNA [16] and its NTR consists of large regions of low-complexity sequence likely to form IDRs (S1A Fig), the full-length AD-Nos fusion does not on its own interact with any component of the “baits” in these four hybrid experiments (see also S4 Data). The specificity of Nos recruitment is further supported by experiments that test alterations at the protein-protein-RNA interface that mediates complex formation between the Nos and Pum RBDs (Fig 1A). Substitutions in the Nos tail (L7) or the NBS essentially abolish recruitment, and the level of reporter transcription upon expression of the Pum[RBD^+^] F1367S mutant is only 6-fold above background (Fig 3C). Taken together, these results are consistent with measurements of Nos RBD recruitment by the Pum RBD in vitro and in our previous qualitative yeast assays [4,5,24].

### The Pum and Nos NTRs potentiate Nos recruitment to NREs in yeast

We next tested whether the Pum NTR has a role in Nos recruitment. As shown in Fig 3C, addition of the NTR has two significant effects. First, it enhances recruitment of full-length Nos to the wt NRE 3.5-fold (fourth column in Fig 3C), even though the NLS fusion to Pum[NTR + RBD] is expressed at a 4.2-fold lower level than NLS-Pum[RBD^+^] (Fig 3D). Second, the Pum NTR ameliorates the effects of substitutions at the RBD-RBD-RNA interface. For example, Nos^L7^ is recruited to a 39-fold lower level than wt Nos by Pum[RBD+], but to only a 3.1-fold lower level by Pum[NTR+RBD] (Fig 3C). The Pum NTR similarly buffers the effects of the F1367S and NBS substitutions in the Pum RBD and the NRE, respectively (Fig 3C). Even with the contribution of the Pum NTR, Nos recruitment is still specific: combining two of the weak substitutions at the protein-protein-RNA interface (in Nos and the NRE), which have a modest effect on their own, reduces Nos recruitment to near-background levels (Fig 3C). Finally, we note that the stimulatory effect of the Pum NTR is completely dependent on the Nos NTR; the Nos RBD (Fig 3B) is recruited to the NRE to the same extent whether Pum bears its NTR or not (ninth column in Figs 3C and S1B).

The NTRs of Pum and Nos consist of large presumptive IDRs (S1A Fig). In other proteins, such IDRs can act as scaffolds that somewhat promiscuously promote interaction with hundreds of proteins [33]. We therefore wondered whether the Pum NTR would be sufficient to recruit Nos to another RNA in the absence of the protein-protein interactions that couple binding of the Nos and Pum RBDs (as in Fig 1A).

To test this idea, we expressed an NLS derivative of the Dm-Ce chimera described above (and shown in Fig 1B), as well as an NLS derivative of the FBF2 RBD alone as a control. We then asked whether the Pum NTR is sufficient to recruit Nos when bound to the wt C. elegans FBE. As shown to the right in Fig 3C, the chimera does not recruit Nos to an appreciable extent: β-galactosidase production is stimulated only 2.4-fold above background by the Dm-Ce chimera bound to the FBE. In contrast, Pum[NTR+RBD], which is expressed to the same level (Fig 3D), recruits Nos and thereby stimulatesβ-galactosidase production 235-fold above background upon binding to its cognate site, the NRE (Fig 3C and S4 Data). Thus, the NTR augments Nos recruitment, but is not sufficient to drive significant Nos binding in the absence of interactions between the Nos and Pum RBDs.

Nos is thought to be the limiting factor for repression of *hb* mRNA in the embryo [34,35]. As described in S1 Text, several lines of evidence support the idea that the level of Nos is also limiting in the yeast four-hybrid experiments reported above. Thus, the yeast experiments appear to appropriately model joint regulation by Nos+Pum in the Drosophila embryo.

A model that summarizes the results of our four-hybrid experiments is shown in Fig 3E. In the absence of the Pum NTR, specific Nos/Pum/NRE complexes assemble via the network of interactions seen in the co-crystal structure [5] and highlighted in Fig 1A. Disrupting any aspect of this network essentially abolishes complex formation, which is driven solely by the Nos and Pum RBDs (i.e., the NBS mutant site on the lower left in Fig 3E). But in vivo, both Nos and Pum possess NTRs, and these collaborate to supplement NRE recognition, perhaps by interacting as in the model. As a result, the ternary complex is stable even if the network of interactions involving the Nos RBD, the Pum RBD, and the NBS is perturbed (i.e., the NBS mutant in the middle of Fig 3E). The Pum NTR appears to have only an auxiliary function in RNA recognition, since on its own, it cannot significantly recruit Nos when tethered to an “inappropriate” FBE site (to the right in Fig 3E), at least when Nos is the limiting factor for complex formation. We note that none of the experiments described above tests the idea that the Nos and Pum NTRs interact with each other, as shown in the figure. This issue is addressed in experiments later in this work.

### The Pum and Nos NTRs potentiate Nos recruitment to NREs in embryos

**Fig 4:**
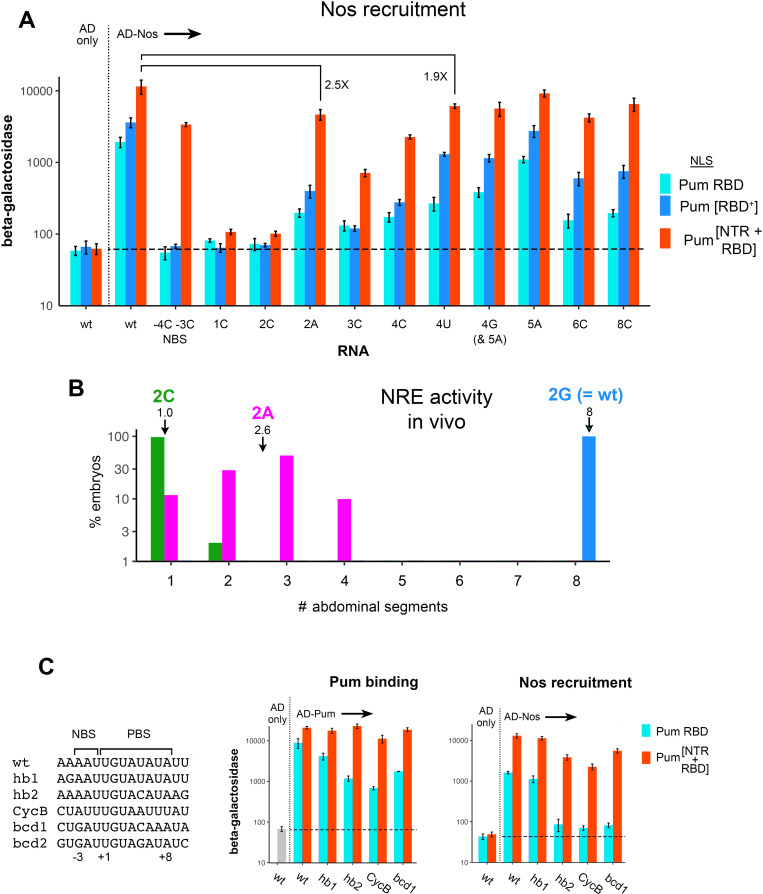
The Pum and Nos NTRs alter binding specificity in yeast to mimic regulation in the embryo. A. Results of four-hybrid experiments measuring recruitment of the AD fused to full-length Nos to the same panel of NREs as in Fig 2C by the three NLS-Pum fusions shown at the right. At the left are AD-only empty vector controls with each NLS-Pum fusion, revealing no significant auto-activation with the wt NRE as part of the bait. Similar experiments were performed with each mutant NRE, showing no significant auto-activation in any case (S4 Data). B. Activity of mutant NREs governing repression of maternal *hb* mRNA in early embryos, as revealed by the extent of abdominal segmentation. For a fuller explanation of the experimental logic, see the text. Embryos from females expressing a maternal *hb* reporter with tandem 2C, 2A, or wt NREs were allowed to develop, and the number of abdominal segments in ≥ 110 embryos was scored. Results are plotted as the % of total embryos for each reporter on the logarithmic y-axis versus the number of abdominal segments per embryo on the x-axis. The 2C and 2A *hb* reporter mRNAs were expressed to the same level (S1 Text and S4 Data). Somewhat confusingly, the NRE was originally defined functionally as a 32-nucleotide element [36] that was later shown to be a composite binding site for Brat, Nos, and Pum. More recently (and throughout this report), the NRE refers only to the Nos+Pum composite site shown in Fig 1A. Brat is essential for full repression of *hb* mRNA [37], and so we test the activity of full 32-nucleotide elements in these experiments. C. With both the Pum and Nos NTRs included, joint binding to various authentic NREs correlates with their activity in vivo. Sequences of the sites tested are shown on the left. Three-hybrid measurements of Pum binding and four-hybrid measurements of Nos recruitment are shown in the center and to the right, respectively. Each site was assayed with the two Pum fragments shown at the right (fused to AD for Pum binding and to NLS for Nos recruitment). The observation that Nos is recruited slightly more efficiently to *hb1* NRE than *hb2* NRE by Pum[NTR + RBD] is consistent with an earlier report showing that the *hb1* site confers somewhat greater repression in embryos [16]. Note that the *bcd* NRE, which was also defined based on functional assays in vivo, is a 45-nt element bearing the two UGUA motifs indicated to the left, *bcd1* and *bcd2* [36]. The individual contribution of each of these to regulation in vivo has not been assessed, to our knowledge. On its own, the *bcd2* NRE does not bind either Pum or Nos+Pum appreciably (S4 Data), although it may contribute to occupancy of *bcd1* NRE in vivo by the mechanism described in experiments described later in this work. Underlying data in S4 Data.

Given our findings that the Pum NTR relaxes binding specificity and stimulates Nos recruitment, we wondered whether these two activities would combine to relax the sequence specificity for Nos recruitment. Such altered specificity would have the effect of broadening the repertoire of sites that potentially mediate regulation by Nos+Pum. To test this idea, we repeated the experiment of Fig 2C (in essence), but measuring recruitment of Nos rather than binding of Pum. The results are shown in Fig 4A.

To a first approximation, Nos binding mirrors Pum binding, with the Pum NTR relaxing specificity to allow efficient Nos recruitment to NREs bearing substitutions in the 3’-proximal portion of the site. This observation is consistent with the unsurprising idea that the primary determinant of site selection by Nos+Pum is binding of Pum. There are three notable exceptions to the overall similarity of the results of measuring binding of Pum and binding of Nos+Pum. First, there is the trivial difference that the NBS site substitutions have no effect on binding of Pum RBD or Pum[RBD^+^] (Fig 2C), but they eliminate Nos recruitment by these Pum fragments (as noted above and shown in Fig 4A). Second, Pum[RBD^+^] appears to recruit Nos more efficiently than Pum RBD to some of the mutant NREs (4U, 4G, 6C, and 8C); in contrast the binding of Pum[RBD+] and Pum RBD to these sites is nearly indistinguishable in the three-hybrid experiments of Fig 2C. This difference is likely due to the 15-fold higher level of NLS-Pum[RBD^+^] than NLS-Pum RBD (Fig 3D).

The third, interesting exception is that the Pum NTR disproportionately relaxes the specificity of Nos recruitment (Fig 4A) to a greater extent than it relaxes the specificity of Pum binding (Fig 2C). This enhanced relaxation by Pum[NTR + RBD] is evident for Nos recruitment to the 2A, 3C, and 4C mutants. In particular, Nos+Pum[NTR+RBD] binds only 2.5-fold less to the 2A mutant NRE than to the wt NRE (Fig 4A); in contrast, binding of Pum[NTR+RBD] on its own to the 2A site is reduced 48-fold (Fig 2C). Indeed, via the combined activities of the Nos and Pum NTRs, Nos is recruited more efficiently to the 2A site than to the NBS mutant site, which has substantial activity in vivo [13]: embryos in which regulation of maternal *hb* mRNA is controlled by NBS mutant NREs develop 2-3 abdominal segments, rather than the full complement of 8 in wt embryos. We realized that the surprisingly high activity of the 2A site in binding Nos+Pum allowed us to test the in vivo relevance of our yeast four-hybrid results, as follows.

Repression of maternal *hb* mRNA in the posterior of the early embryo is essential for the development of abdominal segments. This repression is mediated by two regulatory elements in the 3’-UTR, each bearing a binding site for the Brain Tumor (Brat) co-repressor upstream of a composite Nos+Pum binding site (i.e., the NRE) [36,38]. Previously we have described the use of a maternal *hb* mRNA reporter expressed under native signals in transgenic flies to assay NRE activity [39]. In this *hb* reporter mRNA, the two native regulatory elements that mediate joint regulation by Nos, Pum and Brat are deleted, abolishing repression and thereby blocking abdominal segmentation; adding back a tandem copy of the 3’-proximal element from *hb* completely restores repression and the development of abdominal segments.

Accordingly, we prepared transgenic flies that express the maternal *hb* reporter mRNA with tandem inserts of synthetic elements bearing 2C mutant NREs (as a negative control), wt NREs (as a positive control), and 2A mutant NREs. Translational repression of each reporter was assayed by examining abdominal segmentation among embryos from transgenic females. As shown in Fig 4B, when added back to the maternal *hb* mRNA reporter, tandem wt NREs allow development of a full complement of 8 abdominal segments. In contrast, tandem 2C NREs, which do not bind Nos+Pum above background in yeast four-hybrid experiments, allow development of only 1 abdominal segment, essentially the same phenotype seen in the absence of Nos or Pum function. Strikingly, tandem 2A NREs allow development of 2.6 abdominal segments on average, the same phenotype reported previously for tandem NBS mutant NREs [39]. This degree of abdominal segmentation corresponds to substantial regulation; expression of a *hb* reporter mRNA bearing only a single wt regulatory element confers readily detectable repression of Hb in the posterior of the embryo that is nevertheless insufficient to allow development of any abdominal segments when the *torso*-dependent terminal system is active [36].

The relatively high activity of the 2A mutant NRE is somewhat surprising, since the UGU trinucleotide at positions +1 through +3 of the site is generally considered to be a conserved, invariant signature for the NRE. Indeed, none of the mutants bearing substitutions in the UGU we have tested bind appreciably to the Pum RBD or allow it to recruit Nos in our experiments. But addition of the NTR allows Pum[NTR + RBD] to efficiently recruit Nos to the 2A mutant site in yeast, which correlates with the ability of full-length Pum to recruit Nos and repress the 2A mutant *hb* reporter in embryos. The correlation supports the idea that our yeast four-hybrid experiments faithfully reflect Nos+Pum binding in embryos.

A second test of the in vivo relevance of our yeast four-hybrid results derives from consideration of the well-defined, authentic NREs that have been shown to mediate regulation in vivo of maternal *hb*, *Cyclin B* (*CycB*), and *bicoid* (*bcd*) mRNAs [20,36]. In the experiments described above, we studied binding to a model, pseudo-wt NRE sequence that exhibited high affinity for both the RBD and Nos+RBD in preliminary experiments. The model site is, in essence, a chimera with the NBS of *hb* NRE2 juxtaposed to the PBS of *hb* NRE1 (Fig 4C). We wished to test Pum binding and Nos recruitment to authentic NREs, particularly in view of certain conflicting reports in the literature. For example, the Pum RBD has been shown to bind on its own to the *CycB* NRE in one report but not another [5,20].

Accordingly, we used yeast assays to compare RNA-binding and Nos recruitment to the model pseudo-wt NRE (as a reference) and to the two NREs in *hb*, the *CycB* NRE, and a fragment of the *bcd* NRE (Fig 4C). For each site, we also compared the activity of Pum with and without its NTR. As shown in Fig 4C, with respect to binding to the reference wt NRE, binding of the Pum RBD to the *hb1* NRE is reduced only ~ 2-fold, but is reduced between 5- and 15-fold to the remaining authentic sites. In contrast, binding of Pum[NTR + RBD] to the *hb1*, *hb2* and *bcd1* NREs is indistinguishable from binding to the model NRE, and binding to the *CycB* NRE is reduced < 2-fold. When we assayed the contribution of the Pum NTR to Nos recruitment, the difference in activities with and without the Pum NTR was even more pronounced. The Pum RBD recruits Nos to the *hb1* NRE at 70% the level to which Nos is recruited to the model wt NRE; but recruitment of Nos by the Pum RBD to the remaining authentic NREs is negligible, no more than twofold above background (Fig 4C). This observation is striking, given the well-characterized activity of *hb* NRE2 in embryos [13,36], that underlies the in vivo assays of NRE function in the *hb* reporter experiments described in Fig 4B. In contrast to the poor performance of the Pum RBD, Pum[NTR + RBD] recruits Nos to all the authentic NREs, stimulating β-galactosidase expression between 59- and 179-fold above background (Fig 4C). We conclude that the activity of Pum[NTR + RBD] in these experiments better represents NRE activity in vivo than the activity of the isolated Pum RBD.

In summary, three independent lines of evidence show that the activities of full-length Nos and Pum[NTR + RBD] in the three- and four-hybrid yeast experiments outlined above faithfully reflect the activity of endogenous Nos+Pum in wt embryos. First, maternal mRNAs are targeted by Nos or a Upf1-Nos chimera if they bear either canonical wt NREs or altered 4U NREs [3]. Second, while the NBS and 2A mutant NREs are somewhat less active than the wt NRE in vivo, they have substantial residual ability to repress translation of maternal *hb* mRNA in the embryo (Fig 4B) [13]. Third, the *hb2*, *CycB*, and *bcd* NREs mediate biologically relevant repression by Nos+Pum in vivo [20,36,40]. In all three cases, Pum[NTR + RBD] efficiently recruits Nos to the relevant NREs in our yeast experiments, whereas the isolated Pum RBD does not.

### The Pum NTR interacts with the Nos NTR and with itself

We show above that the Pum NTR acts via the Nos NTR to promote binding of Nos+Pum to the NRE. One mechanism that would account for this aspect of Pum NTR activity involves interaction with the Nos NTR, providing an auxiliary set of protein-protein contacts that augment those made between the Pum and Nos RBDs in proximity to bound RNA (as suggested in the model of Fig 3E).

As stated above, to date, we have not succeeded in purifying soluble fragments of either Nos or Pum bearing large portions of the NTR to test interaction in vitro. We therefore once again turned to yeast to test interaction between Nos and Pum, measuring expression of β-galactosidase encoded by a reporter gene in yeast two-hybrid experiments.

**Fig 5:**
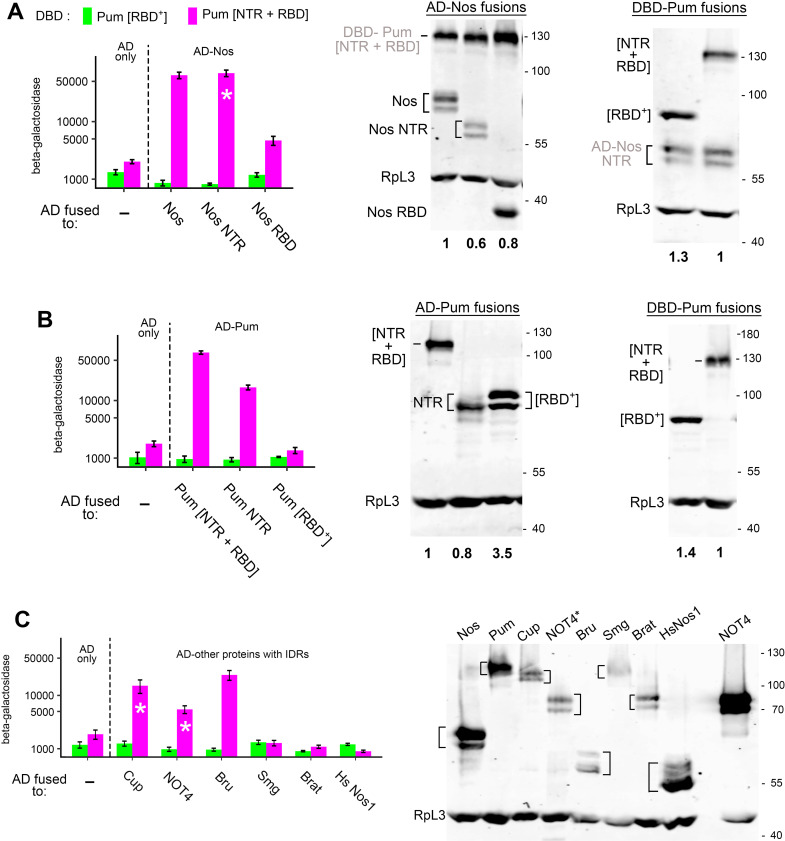
The Pum NTR interacts with itself and with the Nos NTR. A. Yeast two-hybrid experiments reveal interaction between the Pum and Nos NTRs. Throughout the figure, each AD fusion (below the bargraph) was tested with the two DBD fusions shown at the top left. The isolated Nos NTR apparently acts as a serendipitous transcriptional activator, since co-expression of a ΔGAL4 AD fusion to the Nos NTR with DBD-Pum [NTR + RBD] robustly activates transcription (S5 Data). This is consistent with other experiments in which a DBD-Nos NTR fusion autoactivates transcription of the LacZ reporter (S5 Data). In this figure, the three fusions that bear serendipitous transcriptional activation activity are marked with asterisks in the bargraphs. Note that the background level of β-galactosidase in the AD-only controls is higher in the yeast strain used in these experiments than in the strain used for 3- and 4-hybrid RNA-binding experiments elsewhere in this report. Western blots to the right measure the relative levels (in bold below each blot) of the fusion proteins indicated above each blot. On the left, yeast co-expressed a DBD fusion to Pum[NTR + RBD] and on the right, an AD fusion to the Nos NTR, as indicated. In these experiments, Nos derivatives were expressed using the pACT2 vector, since expression using the pGAD vector yielded low levels of protein that are difficult to quantitate reliably (as in Fig 3). B. Similar to A, showing homotypic interaction between Pum NTRs. In the blots to the right, the partner protein co-migrates with one or more of the fusions of interest, and so we prepared extracts from yeast otherwise identical to those used in the β-galactosidase assays, but expressing a partner protein missing the HA epitope tag, DBD-Pum[NTR + RBD] on the left and AD-Pum[NTR + RBD] on the right. C. Similar to A and B, but measuring interaction with other RNA regulatory factors with IDRs. Expression of these AD-fusions (co-expressed with DBD-Pum[NTR + RBD] missing the HA tag) is shown in the blot to the right. We cannot load 3 replicates of each on a single gel, and so it is not possible to rigorously quantitate relative levels. Therefore, the figure displays qualitative differences among expression levels. In the main panel, the NOT4 sample is labelled with an asterisk, to indicate that it consists of a mixture of 10% yeast expressing NOT4 and 90% yeast expressing AD only (so that each lane contains approximately the same amount of protein). An undiluted NOT4 sample is shown in the lane at the far right. Additional Western blots are presented in S2 Fig. Underlying data in S5 Data.

As shown in Fig 5A, interaction between Pum[NTR + RBD] and either Nos or the isolated Nos NTR stimulates the β-galactosidase reporter approximately 33-fold; in contrast, interaction with the Nos RBD stimulates the LacZ reporter only 2.3-fold above background. Interaction depends on the Pum NTR, since none of the Nos fragments interacts detectably with Pum[RBD+].

Most of the mRNAs targeted by Upf1-Nos or Nos in vivo have multiple NREs within their 3’-UTRs, including the canonical Nos+Pum substrate *hb* [3]. This observation led us to wonder whether the NTR might mediate homotypic interactions between NRE-bound Pum molecules that, in theory, could enhance mRNA occupancy and thereby contribute to regulation. As shown in Fig 5B, interaction of Pum[NTR + RBD] with either itself, or with the isolated Pum NTR, stimulates β-galactosidase expression approximately 37- and 9-fold, respectively, in the two-hybrid reporter strain. The interaction is dependent on the NTR in both the DBD-bait and AD-prey fusions, since no interaction is observed if either of the partners in the two-hybrid experiment is a fusion to Pum[RBD^+^].

A major caveat to the results of these two-hybrid experiments is that we do not know whether the observed interactions are direct or indirect (e.g., mediated by bridging yeast proteins). In embryonic extracts, native Pum does not interact detectably with GFP-tagged Nos using sensitive MS methods to identify partner proteins [41], consistent with the idea that Nos and Pum do not form a stable complex. Whether the two-hybrid interactions we observe are direct or indirect, a series of control experiments rule out some of the trivial explanations for the NTR-mediated two-hybrid interactions between Nos and Pum. First, in the experiments of Fig 5A and 5B, the level of the interacting DBD- or AD-fusion is less than or equal to the level of the non-interacting fusions (see also S2 Fig). Second, in no case does interaction significantly alter the relative level of the Nos or Pum partner protein (Figs 5 and S2, and S5 Data). Taken together, our results support the idea that the Pum NTR interacts with itself and with the Nos NTR (either directly or indirectly), but not with either the Nos or Pum RBDs.

An additional concern about interactions mediated by Pum is that some IDRs participate in biomolecular condensates with hundreds (or even thousands) of partners [33]. We therefore wondered whether the Pum IDR might be an unusually promiscuous scaffold. To examine the specificity of interactions mediated by the Pum IDR, we tested interaction with other RNA-binding regulators that have considerable IDRs themselves: four that have been implicated in Nos-dependent regulation (Cup, NOT4, Bru, and Brat) and two that have not (Smg and Hs Nos1). As shown in Fig 5C, three of the four factors that mediate aspects of Nos activity in vivo (Cup, NOT4, and Bru) interact with Pum[NTR + RBD] but not with Pum[RBD^+^], which serves as a negative control. The fourth such factor, Brat, does not interact with Pum[NTR +RBD] (at least with the fragment tested, which includes 388 residues of the Brat NTR). Neither Smg nor Hs Nos1 interacts with Pum[NTR + RBD], even though the fragments tested contain large putative IDRs (approximately 665 and 200 residues, respectively). Thus, the Pum IDR exhibits specificity, interacting with some IDR-bearing proteins but not others.

In summary, the two-hybrid experiments described above reveal interactions of the Pum NTR with itself and with the Nos NTR; it is unclear whether these are direct or indirect. The two-hybrid results are consistent with the model in Fig 3E, in which interactions between the Pum and Nos NTRs off the RNA supplement the network of RNA-proximal interactions between RBDs to stabilize Nos/Pum/NRE complexes. Alternative models are addressed in the Discussion.

### NTRs allow binding to tandem mutant NREs

In all the experiments above, we measured binding to various RNAs, each bearing a single NRE. We wondered whether the hetero- and homotypic interactions between Pum and Nos NTRs might enhance binding to an RNA with multiple suboptimal NREs, which could further expand the repertoire of mRNAs targeted by Nos+Pum.

**Fig 6:**
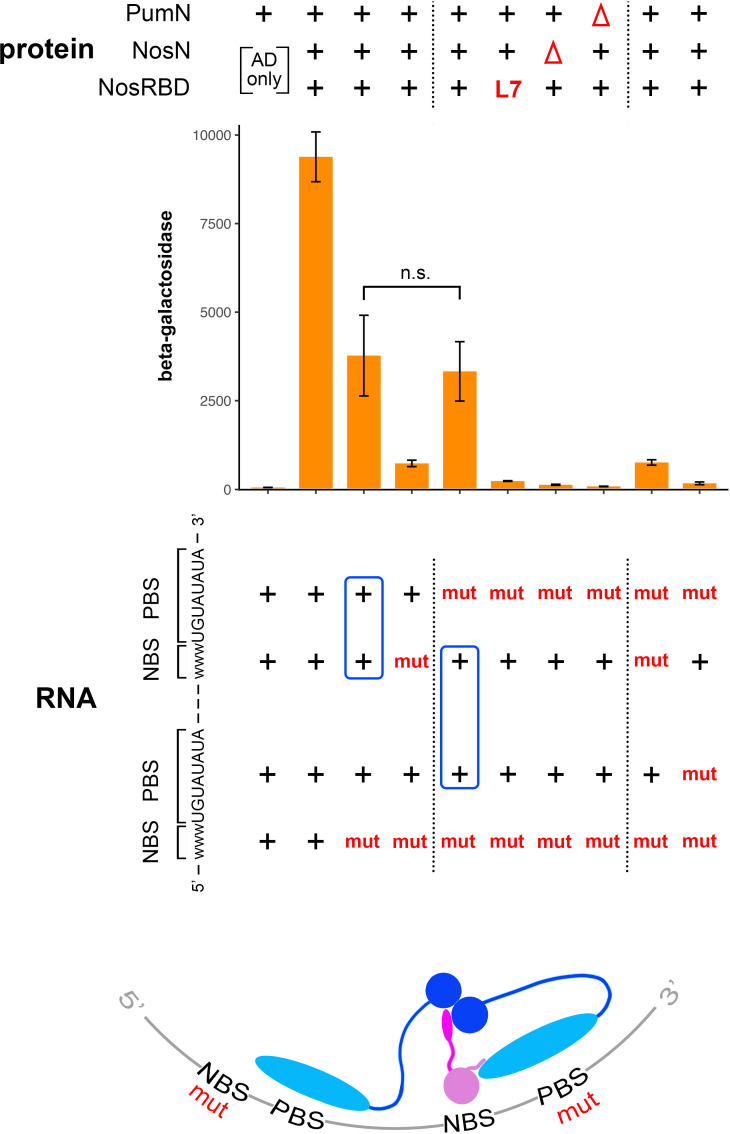
The Pum and Nos NTRs allow recruitment of Nos to an RNA with two suboptimal, mutant NREs. The protein components expressed in each four-hybrid experiment are shown above. The Pum RBD is wt in each case, and the first line indicates whether its associated NTR is present (+) or absent (Δ) (i.e., Pum[NTR + RBD] vs. Pum[RBD^+^]). The second and third lines indicate the identity of the Nos moiety in each case, in particular, whether its NTR is present (+) or absent (Δ) (i.e., full-length Nos or Nos RBD only), and whether its RBD is wild type (+) or bears the L7 mutant. The bargraph is a linear plot of β-galactosidase assays measuring Nos recruitment to RNAs with various tandem NREs. The linear plot is used to avoid compressing the differences among columns 2, 3, and 5, as would occur in a logarithmic plot. Note that the standard deviations appear larger here, but the relative standard deviations are essentially the same in these experiments and in the three- and four-hybrid experiments elsewhere in this report, which are displayed with logarithmic y-axes. The partial sequence of the RNA with tandem wt NREs is below the bargraph to the left, followed by a schematic indication of whether the NBS or PBS element is wt (+) or mutant in each NRE variant. The critical comparison between an RNA with one intact NRE (lane 3) and an RNA with no intact NREs but separated wild type NBS and PBS elements (lane 5) is highlighted in blue boxes. At the bottom is a model for Nos+Pum binding in lane 5. Although shown as a direct interaction between the NTRs, our experiments do not rule out the possibility that bridging yeast proteins mediate an indirect interaction. Further details in the text and in S6 Data, which contains the underlying data.

To test this idea, we returned to yeast four-hybrid experiments to measure recruitment of various AD-Nos fusions to RNAs with tandem NREs (Fig 6). The key result is highlighted in blue boxes below the bargraph: when both Nos and Pum proteins possess their respective NTRs, Nos is recruited to essentially the same extent to (1) an RNA with a single wild type NRE (column 3) as to (2) an RNA with no wildtype NREs, but one in which tandem mutant NREs supply non-adjacent binding sites for Pum and Nos (column 5). Nos recruitment to this RNA with tandem mutant NREs is relatively efficient, with β-galactosidase levels 35% of those for recruitment to the control RNA with tandem wildtype NREs. Furthermore, Nos binding to the tandem mutant NREs is dependent on both the Pum and Nos NTRs, the C-terminal Nos tail that mediates interaction between the Nos and Pum RBDs, and the presence of a wildtype NBS and PBS on the RNA.

One interpretation of the experiments is shown at the bottom of Fig 6. In essence, Pum bound upstream can drive formation of a downstream Nos/Pum/RNA complex “at a distance,” even when the downstream 3C mutant site has only a low affinity for Pum (as shown in Fig 2). Although we favor this model, we cannot rule out an alternative in which a novel deployment of the Nos C-terminal tail, mediates interaction between Pum bound to the upstream PBS and Nos bound to the downstream NBS.

We note that the key RNA sequence in these experiments (used in columns 5-8 of Fig 6) would not emerge from a bioinformatic analysis as a likely target for Nos+Pum, since it does not have an intact NRE. The results of Fig 6 suggest that the NTRs of Nos and Pum may expand the repertoire of mRNA targets beyond our current understanding, although we have not yet tested whether such sequences can mediate regulation in vivo.

### The human Pum NTRs share some properties of the Drosophila Pum NTR

**Fig 7:**
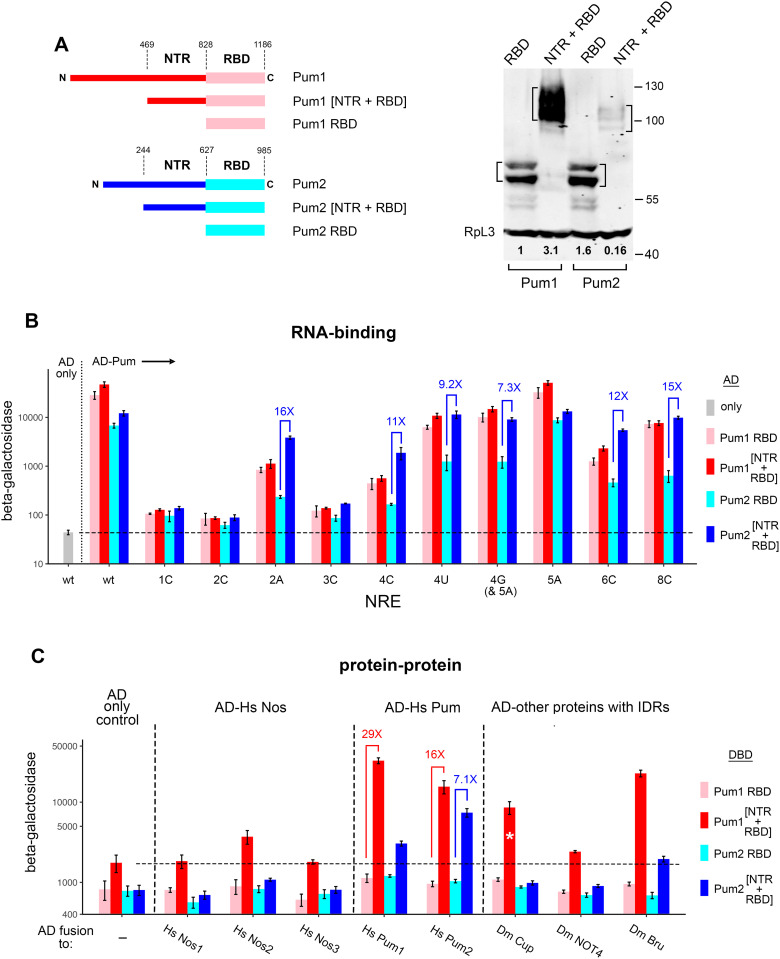
The human Pum2 NTR alters RNA-binding but the Pum1 NTR does not. A. Drawing to scale of the full-length human Pum1 and Pum2 proteins, as well as the [NTR + RBD] and RBD fragments used in the experiments below. To the right are Western blots showing the levels of these fragments, relative to the Hs Pum1 RBD, in bold below. Unlike the Drosophila proteins tested above, proteins with the Hs Pum1 or Hs2 Pum NTR appear to be either multiply modified or partially degraded in yeast. See also S3 Fig. B. Binding of the AD-Pum fusions indicated at the right to various derivatives of a *hb* NRE in yeast three-hybrid experiments, as in Fig 2C. For Pum2, the enhancement of RNA-binding by its NTR is emphasized by labelled brackets (i.e., “16X = 16-fold enhancement). C. Yeast two hybrid experiments testing interaction of the four DBD-fusions indicated on the right to AD-fusions indicated below the bargraph. Significant interactions between Pum NTRs are highlighted with brackets, as above. The white asterisk indicates that Dm Cup bears sequences that fortuitously activate transcription independent of the GAL4 AD, as described in Fig 5 (and S5 Data). Underlying data in S7 Data.

We next asked whether the NTRs of Hs Pum1 and Hs Pum2 have activities similar to those described in Fig 2 through Fig 5 for Drosophila Pum. Preliminary experiments revealed that residues near the N-terminus of each human protein auto-activate transcription of LacZ reporters in yeast assays (S2 Data), and so we confined our analysis of the human Pum NTRs to the RBD-proximal portion of each. The human Pum NTRs studied in this report are approximately the same size as the NTR in Drosophila Pum[NTR + RBD] (Fig 7A).

The RBDs of the human proteins share 75% identity with the Drosophila RBD (and 90% identity with each other); in addition, they have identical RNA base-contacting residues and bind to the same PBS consensus site in vitro as the Drosophila RBD [5,19]. In contrast, the NTRs of the human proteins consist of low complexity sequence predicted to form IDRs (like the Drosophila protein), with four amino acids-- Ala, Gly, Gln, and Ser-- making up approximately 54% of each of the three NTRs. Excluding low-complexity regions, the Hs Pum1 and Hs Pum2 NTRs share only 7.4% and 17% identity with the Drosophila NTR, respectively (and 32% with each other).

We next asked whether the human Pum1 and Pum2 NTRs share three properties attributed to the Drosophila NTR in experiments described above: (1) alteration of RNA-binding via the RBD in cis; (2) interaction with human Nos proteins and promotion of their recruitment into ternary Nos/Pum/NRE complexes; (3) homotypic interaction with each other.

First, we measured binding of Hs Pum1 and Hs Pum2 in three-hybrid experiments, with and without the NTR for each, to the panel of mutant NREs used in experiments with Drosophila Pum. As shown in Fig 7B, Hs Pum2 behaves in a manner similar to Drosophila Pum; inclusion of its NTR enhances binding to the wt NRE and significantly relaxes specificity for the 2A mutant site as well as for sites bearing substitutions at positions +4, +6, and +8. Enhanced binding is not due to the expression of higher levels of Hs Pum2[NTR + RBD], which is actually expressed at a 10-fold lower level than Hs Pum2 RBD (Fig 7A). In contrast, the NTR of human Pum1 has essentially no effect on RNA-binding (Fig 7A and S7 Data).

Second, we asked whether the human Pum NTRs mediate interaction with any of the three human Nos proteins in yeast two-hybrid experiments. As shown in Fig 7C, they do not; the only minor exception is a weak interaction between Hs Pum2 [NTR + RBD] and Hs Nos2, which stimulates β-galactosidase expression 2-fold above background. The failure to observe interaction with Hs Nos1 is particularly striking, as it is expressed at a relatively high level (S3E Fig) and bears a 212-residue NTR that is predicted to consist primarily of IDR. Despite the absence of detectable two-hybrid interactions between the human Pum and Nos proteins, cooperative binding to the RNA might nevertheless allow recruitment of Nos into ternary complexes, as has been described for the Drosophila proteins. However, we see no evidence of recruitment of any of the human Nos proteins by either Pum protein in yeast four-hybrid experiments analogous to those reported above for Drosophila Nos+Pum (S7 Data).

Third, we asked whether the human Pum NTRs interact with each other in yeast two-hybrid experiments. As shown in Fig 7C, the Hs Pum1 NTR mediates (direct or indirect) interaction with itself and with the Hs Pum2 NTR, whereas the Hs Pum2 NTR is more selective, mediating interaction only with itself. We further tested the specificity of these IDR-mediated interactions, by measuring binding to the three Drosophila factors that interact with the Drosophila Pum NTR. As shown in Fig 7C, Hs Pum1[NTR + RBD] interacts with Dm Cup and Dm Bru, but not Dm NOT4; Hs Pum2[NTR + RBD] does not interact significantly above background with any of the Drosophila factors.

In summary, the human and Drosophila Pum proteins share some but not all of the properties described in this report. Previous work has shown that the Hs Pum1 and Pum2 RBDs have indistinguishable binding specificity and affinity [19]. Our results suggest that, with their respective NTRs in cis, the two proteins have different binding site specificity and affinity; they may interact differently with various protein partners as well. A major caveat is that we have only tested a fragment of each Hs Pum, and the full-length, endogenous proteins or other isoforms may behave differently (as discussed further below).

### NTR-dependent binding of a Drosophila Pum mutant with altered sequence selectivity in yeast

In the yeast three- and four-hybrid experiments described above, the main effect of the Pum NTR is to enhance binding to a variety of NRE mutants that have low but detectable affinity for Pum. For example, the wild type Pum RBD binds weakly to the 4U mutant NRE, and addition of the NTR enhances binding 12-fold (Fig 2C). In work to be described elsewhere, we have isolated mutant derivatives of the Pum RBD that use non-wild type amino acids to recognize various NREs. One of these proved to have the interesting property of NTR-dependent binding to the wt NRE, as described below.

Mutants with altered sequence selectivity were identified in Pum[NTR(Δ2) + RBD]. This deletion derivative bears the 5’-proximal half of the Pum NTR and retains the ability to enhance binding to mutant NREs (S4 Fig). For example, binding of Pum[NTR(Δ2) + RBD] is indistinguishable from binding of Pum[NTR + RBD] to the 2A, 4U, and 4G NREs (S4 Fig). In contrast, a deletion derivative bearing the 3’-proximal half of the NTR, of Pum[NTR(Δ1) + RBD], exhibits binding specificity similar to that of the RBD (compare Figs 2C and S4).

**Fig 8:**
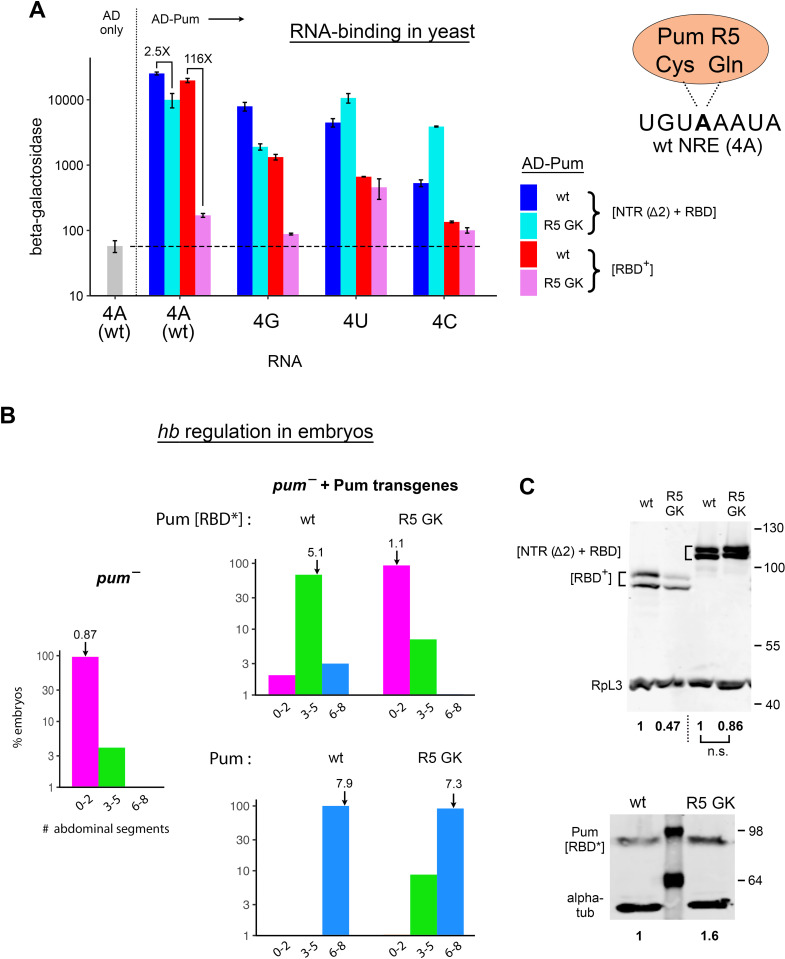
The Pum NTR is essential for activity of a RBD variant in yeast and in embryos. A. The results of yeast three-hybrid experiments measuring the binding of the four proteins indicated at the right to four NREs, each bearing a different base at position +4, as indicated beneath the bargraph. Pum binding is modular, with each Puf repeat making contact to a single base of the NRE. The fifth repeat (R5) of the wt protein uses Cys and Gln residues to preferentially recognize the 4A NRE, as shown schematically to the right. The R5 GK mutant bears Gly and Lys residues at the RNA-contacting positions in R5 and is otherwise wt. In these experiments, each RNA bears an A rather than a U at position +5 to avoid creation of a potential cryptic Pum binding site in the 4G mutant. B. Pum-dependent regulation of maternal *hb* mRNA governs abdominal segmentation. Abdominal segmentation was scored among embryos from *pum* mutant females (left) or *pum* mutant females that carry one of four transgenes, as indicated (panels on right). Transgenes on the top row encode wt or R5 GK mutant Pum[RBD*], a fragment similar to Pum[RBD^+^] (see S4 Fig). Transgenes on the bottom row encode either wt or R5 GK mutant full-length Pum, as indicated. Abdominal segmentation is displayed as in Fig 4B, but with embryos binned as indicated on the x-axis of each bargraph. C. The Western blot above shows the relative expression level in yeast of the four proteins in the experiments of A. The Western blot below shows the relative expression level (in bold below) of wt and R5 GK mutant Pum[RBD*] in 0-2 hr embryos. Transgenic Pum proteins were Myc-epitope tagged near the C-terminus and detected with anti-Myc antibody, with endogenous α-tubulin as a loading control. Underlying data in S8 Data.

The fifth Puf repeat (R5) of the wild type RBD uses Cys and Gln residues to make sequence-specific contacts to 4A in the wild type NRE, as shown in Fig 8A. We initially isolated a mutant bearing Gly and Lys at the base-contacting residues of the fifth repeat (hereinafter, the GK mutant) in a screen for proteins that bind the 4U site. Subsequent analysis revealed that the R5 GK mutant also recognizes the wt 4A NRE efficiently, with binding reduced only 2.5-fold in comparison to the wt protein (Fig 8A). Overall, the wt and GK mutant of Pum[NTR(Δ2) + RBD] proteins exhibit different sequence selectivity, with the wt binding 4A> 4G> 4U>> 4C and the GK mutant binding 4U = 4A> 4C> 4G (Fig 8A).

We also tested binding of an R5 GK derivative of Pum[RBD^+^], which led to a remarkable observation: binding of R5 GK to the wild type (4A) NRE is almost completely dependent on the NTR. As shown in Fig 8A, in the absence of the NTR, binding of R5 GK to the 4A site is reduced 116-fold in comparison to wt Pum[RBD^+^]. Accumulation of the R5 GK mutant Pum[RBD^+^] is reduced 2-fold relative to wt (Fig 8C). However, this small difference seemed unlikely to account for the > 100-fold reduction in binding. Therefore, we proceeded to test activity of the mutant protein in vivo, as follows.

### NTR-dependent regulation of *hb* mRNA by the Pum GK mutant in embryos

As described above for the experiments of Fig 4B, joint regulation of maternal *hb* by Nos+Pum is necessary and sufficient to allow abdominal segmentation [42–44]. Regulation is mediated by two NREs in the *hb* 3’-UTR, each of which bears a canonical A at position +4 (sequences shown in Fig 4C). In embryos from females bearing a *pum* hypomorph, maternal *hb* is not repressed and the embryos develop on average < 1 abdominal segment (Fig 8B). In this genetic background, previous work has shown that abdominal segmentation is partially rescued by maternal expression of a C-terminal fragment of the protein similar to Pum[RBD^+^] that lacks the NTR (here named Pum[RBD*], see S4A Fig), and completely rescued by expression of full-length wild type Pum [13]. Consistent with these studies, we find that wild type Pum[RBD*] represses the endogenous wt *hb* mRNA sufficiently to rescue development of 5.1 abdominal segments on average (compared to the full complement in wt embryos of 8), and full-length Pum rescues development of 7.9 abdominal segments on average (Fig 8B).

The properties of the R5 GK mutant in yeast binding experiments make a clear prediction: it should rescue the abdominal segmentation defects of *pum*^-^ embryos, but only with the Pum NTR in cis. As shown in Fig 8B, we do indeed observe NTR-dependent activity of the R5 GK mutant in vivo. In the absence of the NTR, the R5 GK mutant does not significantly rescue abdominal segmentation above the low level in the *pum* mutant background (*p* = 0.18 by Fisher’s Exact Test). In contrast, with the NTR in cis (as part of the full-length protein), R5 GK drives almost complete abdominal segmentation (7.3 segments on average), only slightly less than the 7.9 segments (on average) in embryos rescued by full-length Pum with a wild type RBD. The R5 GK mutant derivative of Pum[RBD*] is expressed to a slightly higher level (1.6-fold) than wild type Pum[RBD*] in early embryos (Fig 8C), and thus its inactivity is not due to reduced expression or stability. Finally, we note that although the full-length GK mutant efficiently rescues abdominal segmentation, most of the resulting larvae are inviable, with few surviving to third instar (< 1%). In contrast, the majority (> 50%) of *pum*^-^ embryos rescued by the wt full-length control protein survive to third instar or beyond. A likely explanation is that the GK mutant has altered binding specificity (as shown in the yeast experiments of Fig 8A), and therefore inappropriately represses a number of mRNAs in embryos, such as those bearing 4C NREs.

In conclusion, the Pum NTR likely promotes regulation of *hb* in the embryo by the R5 GK mutant via both of the activities identified in this report-- (1) altered recognition of NREs by the Pum RBD in cis and (2) stabilization of Nos+Pum binding to the NRE.

## Discussion

Previous work on RNA recognition by Pum and Nos has focused on studies of their RBDs. In this report, we show that the NTRs of both proteins play important roles in RNA binding and discuss models for how they may act.

We have made use of yeast experiments to measure RNA-binding and protein-protein interactions for a variety of Nos and Pum fragments. We have also performed three independent tests of Nos+Pum function in embryos (Figs 4B, 4C and 8B), where *hb* regulation is mediated by full-length proteins. In these tests, the behavior of NTR-bearing Pum fragments in yeast recapitulates the activity of the full-length proteins in vivo; in contrast, the behavior of the isolated Pum RBD in yeast does not. Pum[NTR + RBD] is missing the N-terminal 644 residues of the major protein isoform (PA) in early embryos, and thus it is perhaps somewhat surprising that its activity in yeast correlates so well with activity of full length Pum in embryos. But three of the other four known Pum isoforms consist of a nearly identical NTR (as defined in Fig 1B) fused to the same RBD; only the PB isoform bears a portion of the 644 residue N-terminal “extension” present in the PA isoform. Perhaps, then, the essential functions of Pum, at least for NRE recognition and Nos recruitment, are embedded in Pum[NTR + RBD]. The N-terminal extension might play other roles in regulation, such as interacting with Brat, which binds adjacent to the *hb* NREs [38,45].

How might the Pum NTR act in cis to alter RNA-binding? One simple possibility is that the NTR harbors an accessory motif that makes sequence non-specific contacts to RNA outside the NRE. We have not yet systematically looked for evidence of direct binding to RNA of the NTR, which bears none of the well-characterized RNA-binding motifs. Sequence non-specific contacts could account for the dramatically enhanced apparent affinity of the chimera bearing the Drosophila Pum NTR fused to the C. elegans FBF2 RBD; despite accumulating to a 9-fold lower level than the FBF2 RBD, the chimera binds to a 9-fold higher level (Fig 2). Several precedents for this sort of coupling between RNA-binding motifs in cis have been described. For example, on its own, the first RRM of Xenopus PABP does not bind detectably to poly(A) and the second binds with only modest affinity [46]. However, a fragment of PABP that contains both RRMs binds with high avidity, as does a similar fragment of the closely related human PABP [47]. Another example comes from the fused in sarcoma (FUS) RNA-binding protein, which uses sequence non-specific contacts made by its short Arg-Gly-Gly (RGG) motif to significantly increase binding mediated by zinc finger and RRM domains elsewhere in the protein [48].

Sequence non-specific binding by some part of the NTR would raise the affinity of Pum[NTR + RBD] for all NRE variants, possibly causing an apparent change in specificity. For example, at the concentration of protein expressed in yeast, sites with moderately lower intrinsic affinity for the isolated RBD (e.g., the 4U, 4G, 6C, and 8C NREs in Fig 2C) would be occupied by Pum[NTR + RBD], while those with much lower intrinsic affinity for the RBD (e.g., the 1C and 2C NREs) would not.

Another model for how the Pum NTR might alter binding of its RBD in cis involves allosteric regulation of the RBD. Such regulation might involve contacts between the NTR and RBD. Although we see no evidence of such contacts in yeast two-hybrid experiments (Fig 5), they might be weak and thus undetectable unless the two domains are tethered to each other. In fact, allosteric regulation of the Pum RBD has already been described: when it binds jointly with the Nos RBD to the NRE, the affinity of the Pum RBD is increased but the sequence selectivity at NRE positions +5 through +8 is decreased [5]. In the crystal structure of the Nos RBD/Pum RBD/NRE complex, interactions of Nos with positions -3 to -1 of the NRE and with the C-terminal end of the Pum RBD cause local conformational changes in the protein. Pum remains in contact with bases +5 through +8 in the NRE, and it is currently unclear how specificity at these positions is relaxed. Another example of allosteric regulation comes from recent work on C. elegans FBF2 and its LST-1 cofactor [31,49]. FBF2 binding is auto-inhibited by a disordered region of its C-terminal tail, which interacts with residues on the “outside,” non-RNA contacting surface of the RBD. In turn, a short, disordered portion of LST-1 can competitively displace the tail, simultaneously relieving inhibition and relaxing binding specificity for bases at the 3’-end of the binding site.

Our experiments reveal that the Pum NTR has another activity, in addition to modulating binding of Pum: in concert with the Nos NTR, it stimulates joint binding of Nos+Pum to the NRE (Fig 3). In principle, the Nos and Pum NTRs could act independently of each other by either of the two mechanisms outlined above, supplying accessory binding energy with sequence non-specific contacts outside the NRE or via allosteric regulation of their respective RBDs. The observation that the Nos and Pum NTRs interact in yeast two-hybrid experiments (Fig 5) leads us to favor a third model, in which weak NTR-NTR interaction off the RNA raises the avidity of Nos+Pum, increasing the probability of joint occupancy by both RBDs (Fig 3E). We believe the NTR-NTR interaction to be relatively weak, since neither we nor others [41] have shown co-immunopurification of native Nos and Pum from embryonic extracts. The two-hybrid interaction might be mediated by intervening yeast factors, particularly since both NTRs are intrinsically disordered, which could drive interaction via bridging proteins. However, it seems unlikely that fortuitous, indirect interactions in yeast would mimic the activity of the Nos and Pum NTRs in Drosophila embryos so accurately. But in the absence of evidence that purified protein fragments interact in vitro, we cannot exclude the possibility that the NTRs interact indirectly in yeast (or in embryos). One possible route to defining the molecular mechanism of action for the Nos and Pum NTRs would be to identify small regions of each that are sufficient to stimulate binding and can be modelled by synthetic peptides. Such an approach has been successful in studies of Nos interaction with the CCR4/NOT/deadenylase complex and of C. elegans LST-1 with FBF2 [21,31].

We note that both the inter- and intra-molecular activities of the Pum NTR likely expand the repertoire of regulated mRNAs beyond those bearing canonical NBS+PBS sequences. As described above, relaxation of binding specificity mediated in cis by the Pum NTR appears to account for the efficient targeting of 4U “mutant” NREs by Nos and Pum in vivo [3]. In Fig 6, we show that Pum can recruit Nos relatively efficiently to an RNA with no intact NRE in yeast, as long as the NTR of each protein is present. It will be important in future to test whether such RNA sequences mediate regulation in vivo with endogenous proteins.

In addition to its effects on RNA-binding, the Pum NTR may act as a scaffold for protein-protein interactions that regulate mRNA translation and stability, based on the results with Cup, NOT4, and Bru shown in Fig 5. The structure prediction program alpha-fold [50,51] identifies only one feature in the otherwise unstructured Pum NTR: a 35 residue alpha-helix consisting of residues 711-745 that is predicted with low- to moderate confidence. The Pum NTR may therefore promote interaction with itself, with similarly disordered regions in the NTR of Nos, and with various translational regulators (e.g., Cup, NOT4, and Bru) via liquid-liquid phase separation, rather than via distinct binding motifs for each partner. It is possible that the RNA bound to Nos+Pum also contributes to aggregation, as has been suggested previously for RNA-binding proteins bearing aggregation-prone domains [52]. Two other reports are consistent with these ideas. First, the Drosophila Pum NTR has been shown to aggregate when expressed in yeast [53]. And second, when expressed in HeLa cells, the Hs Pum2 NTR drives the protein into stress granules, which are phase-separated RNA-protein condensates [54].

The human and fly Pum proteins share a number of structural and functional features, consistent with the idea that they have similar roles in vivo. Each consists of a relatively large N-terminal extension, which is likely disordered, and a highly conserved Puf repeat RBD. The RBDs are structurally similar and bind the same consensus RNA sequence with indistinguishable site specificity and affinity [5,19,32]. These observations have led to the general idea that functional differences between Hs Pum1 and Hs Pum2 are driven by interaction with different protein partners or different subcellular localization, for example. The results of Fig 7 support the idea that their NTRs mediate different protein-protein interactions, with themselves and potentially with other cofactors. The results of Fig 7 further suggest their NTRs may endow different binding specificity and affinity on the two proteins as well. Although this is perhaps the most intriguing idea to emerge from our studies of the human Pum proteins, a significant caveat is that we have not tested activity of the entire N-terminal extension of either protein. In addition, there are many isoforms of both Hs Pum1 and Pum2 that have different N-terminal extensions, which might endow different properties.

At first glance, the structural similarities of the Drosophila and human Nos proteins suggest they might act in a similar manner too. Each consists of an IDR fused to a Zn-finger RBD that has a similar sequence and likely a similar structure [55]. But what is currently known about the function of three Hs Nos proteins suggests their mechanism of action is somewhat different from that of Drosophila Nos. All four proteins interact with components of the CCR4/NOT/deadenylase complex, but the human and fly Nos proteins interact via different motifs [21]. Furthermore, to our knowledge there is neither compelling genetic nor biochemical evidence for joint activity of the human Nos and Pum proteins. The Zn finger domains of human Nos2 and Nos3 have been reported to bind jointly with human Pum1 and Pum2 to RNA in vitro, but at concentrations of approximately 10 µM where non-specific interactions with either protein or RNA (or both) are difficult to exclude [56].

It is therefore perhaps not surprising that we see evidence of neither interaction between the human Nos and Pum proteins (Fig 7C) nor joint recruitment to the NRE in four-hybrid experiments (S7 Data). Of course, these are negative results, subject to the further caveat that only a portion of a single isoform of each human Pum protein was tested. Regulation of some target mRNAs by murine Nos2 has been shown to be mediated by the RRM protein DND1 in the male germline [57]. Thus, there exists at least one Pum-independent mechanism to recruit human Nos2 to RNA. Further work will be required to determine whether the full-length human Pum proteins play any role in Nos recruitment, or in target site selection and mRNA regulation in vivo.

## Materials and Methods

### Drosophila strains and methods

Fly stocks were maintained by standard methods and grown at 25°C. Transgenic lines were constructed by microinjection of *w*^1118^ embryos using standard methods. For the experiments in Fig 4B, abdominal segmentation was scored both in otherwise wt flies and in *hb*^FB^/ + heterozygotes. Abdominal segmentation is sensitive to the level of maternal *hb* mRNA, as reflected in the slight difference in the average number of segments in the two backgrounds (S4 Data). We did not score embryos with the zygotic *hb*^FB^/ *hb*^FB^ phenotype, which has confounding posterior defects and can be readily identified by characteristic head and thoracic defects. Although we did not measure expression of the *hb* reporter bearing tandem wt NREs, it substantially rescues the zygotic *hb*^FB^/ *hb*^FB^ phenotypes and thus is presumably expressed to normal levels. For measurement of transgene-encoded *hb* reporter mRNA, total RNA was prepared by homogenization of 0-1 hr embryos. cDNA was then synthesized from 250 ng RNA using random hexamers and qPCR performed using SYBR Green and a 7500 Real Time PCR machine (Applied Biosystems). Primers were designed to hybridize specifically to transgenic mRNA; in control experiments, these direct the synthesis of approximately 1000-fold more qPCR product with cDNA from transgenic embryos than from control embryos, in which only endogenous wt *hb* mRNA can serve as template (S4 Data). Triplicate measurements were obtained for each of three biological replicates. Abundance of each RT product was normalized to the abundance of RT product from RpS2 mRNA. For the experiments in Fig 8, *pum*^-^ flies were trans-heterozygotes of *pum*^Msc^ and *pum*^ET3^. We observed only minor differences among three or more transgenic lines, which were tested in preliminary experiments for rescue of abdominal segmentation. Embryos were collected on apple-juice agar plates smeared with yeast paste. Embryonic cuticle was examined by harvesting embryos 24 hours after removing adults, dechorionating with bleach, and mounting in Hoyers/lactic acid. Slides were cleared by heating for several hours and then examined by dark field microscopy using a Zeiss Axiophot. For the Western blot of maternal proteins in Fig 8, 0-2 hr embryo collections were homogenized in sample buffer, electrophoresed on a SDS denaturing gel, and transferred to 0.2 µm nitrocellulose membranes by standard methods. Four biological replicates of each genotype were analyzed on a single blot. The epitope-tagged Pum and α-tubulin proteins were detected by incubation with anti-Myc 9E10 monoclonal primary (SC-40, Santa Cruz Biotechnology) and anti-α-tubulin B-5-1-2 monoclonal primary (T5168, Sigma), followed by goat-anti mouse IRDye 680 goat anti-mouse IgG secondary antibodies (926-68070, LiCor), and detected on a LI-COR Odyssey CLx. Bands were quantitated using LiCor Image Studio software.

### Plasmids

Plasmids used in this work and descriptions of their construction are in S9 Data. The protein expressed in flies that is identified here as Pum[RBD*] is identical to Δ2’ in [13] with the exception of a 6X-Myc epitope tag near the C-terminus. The Pum[RBD*] ORF begins at the native Pum AUG initiation codon and continues through the first 27 residues of the PA isoform, which are fused to the C-terminal 442 codons encoding the RBD and C-terminal residues. The protein identified here as the Nos RBD is identical to Nos ΔN1 in [4] and bears, in addition to the minimal Nos RBD, the N-terminal 42 residues of Nos, which are required for efficient expression of the RBD in yeast for unknown reasons. We experienced difficulty manipulating Hs Nos1 sequences encoding its NTR, likely due to their high (approximately 80%) G+C content. We bypassed this issue and simultaneously optimized expression in S. cerevisiae using the Odysseus program [58] to design a new NTR-coding sequence. Sequence of the modified Hs Nos1 gene is in S9 Data. A cDNA clone encoding FBF2 was prepared by reverse transcription of RNA prepared from mixed stage N2 C. elegans. For expression of RNA in 3- and 4-hybrid experiments, sequences inserted into pIII/MS2-2 [18] were analyzed via m-fold [59] to ensure accessibility of each binding site.

### Yeast experiments

Measurements of β-galactosidase were performed essentially as described [3], from which the following description is taken. Yeast were transformed with the 2µ plasmids described in S9 Data by a standard lithium acetate/PEG protocol. For 2-hybrid experiments, we used the PJ69-4A strain [60], which is *MAT****a***, *trp1-901*, *leu2-3*, *112*, *ura3-52*, *his3-200*, *gal4*∆, *gal80*∆, *LYS2::GAL1-HIS3*, *GAL2-ADE2*, *met2::GAL7-lacZ*. Double transformants were obtained on minimal SD dropout medium lacking tryptophan and leucine. For 3- and 4-hybrid experiments, we used the YBZ1 strain [26], which is *MAT****a****, ura3-52, leu2-3, 112, his3-200, trp1-1, ade2, LYS2::(LexAop)-HIS3, ura3::(lexA-op)-lacZ, LexA-MS2 coat (N55K)*. For 3-hybrid experiments, double transformants were grown in minimal SD dropout medium lacking uracil and leucine, and for 4-hybrid experiments triple transformants were grown in SD dropout medium lacking uracil, leucine, and tryptophan.

β-galactosidase activity was measured using β-Glo (Promega) essentially as described [26]. Briefly, transformants were grown by diluting saturated overnight cultures into appropriate selective media to early-log phase (OD_600_ ~ 0.3), and then 40 µl of culture were incubated with the same volume of β-Glo reagent for 60 minutes at room temperature in 96-well microplates. Four transformants were grown separately and assayed for each experiment, occasionally omitting a culture that grew unusually slowly or quickly. Samples were analyzed in a Veritas luminometer (Turner Biosystems Inc). The output signal from the luminometer was divided by the OD_600_ to normalize for the number of cells in each sample, and by 1000 (by convention) to generate a reading of β-galactosidase activity in arbitrary light units. *p*-values reported in Supporting Data are from unpaired t-tests. Experiments for each figure panel were performed at the same time to avoid variability due to minor fluctuations in reaction conditions or substrate activity.

Western blot samples were prepared from yeast cultures grown in selective media to OD_600_ = 0.3, and then processed essentially as described [61]. Cells were harvested by centrifugation and resuspended in 200 µl of a solution containing 0.1 M NaOH, 0.05 M EDTA, 2% SDS, 2% β-mercaptoethanol, and 8M urea. After a 10 min incubation at 70º, samples were neutralized with the addition of 5 µl of 4M acetic acid and vortexed for 30 s. Aliquots were stored at -80º and briefly reheated at 70º before SDS-PAGE. In any experiment where proteins of interest co-migrate, we used expression of an otherwise identical derivative of one of the fusions that lacks the HA epitope tag. Western blots were performed as described above, using monoclonal C29F4 rabbit anti-HA (3274, Cell Signaling Technology) to detect plasmid-encoded proteins tagged with the HA epitope and mouse monoclonal Sc Rpl3 (Developmental Studies Hybridoma Bank) to detect RpL3 (as a loading control). The anti-mouse secondary antibody from LiCor is described above, and the anti-rabbit IRDye 800 goat anti-rabbit IgG secondary antibody was 926-32211 from LiCor. Each blot contained either three or four biological replicate samples. To generate values for the relative expression of various proteins, the bands from a single blot were quantitated using LiCor Image Studio software. *p*-values reported in Supporting Data are from unpaired t-tests.

## Supporting information

S1 Fig Nos recruitment to the NRE in yeast four-hybrid experiments. A. Plots of the probability of disorder calculated using IUPred2A [62] (y-axis) for Pum[NTR + RBD] and Nos, plotted versus residue number of the full-length proteins on the x-axis. B. Similar to the experiment of Fig 3C, but comparing Nos recruitment by Pum[NTR + RBD] and by Pum RBD. Note that the level of NLS-Pum[NTR + RBD] is higher than the level of NLS-Pum RBD (Fig 3D); this may contribute somewhat to the higher level of LacZ reporter activity for the former protein, although we argue in the text that the level of AD-Nos (rather than the level of NLS-Pum) is the primary limiting factor in these experiments. Experiments with the FBE are the same as in Fig 3C, shown for comparison to the results of over-expressing AD-Nos in C, as follows. C. A subset of the experiments in B, but showing recruitment of Nos expressed from pACT2 plasmid derivatives that direct the expression of higher levels of protein, as shown in D. D. Relative Nos expression levels (in bold below), reveal that Nos is expressed to an approximately 20-fold higher level in the experiments of C compared with the experiments of B (or Fig 3C). Note that AD-Nos and NLS-Pum[NTR + RBD] are approximately equimolar (S3 Data). There is no significant difference in accumulation of wt Nos and Nos^L7^, consistent with the observation that the mutant protein is stable in embryos [63]. Underlying data in S3 Data.
(TIF)

S2 Fig Expression levels of the DBD- and AD-fusion proteins used in the two-hybrid experiments of Fig 5. Each panel is a Western blot to measure the relative levels of proteins named above the image and quantitated below in bold. All samples are from yeast co-expressing the relevant factors shown in the two-hybrid experiments of Fig 5. In A, C, and D, where AD- and DBD-fusion proteins comigrate, yeast co-expressed an otherwise identical but untagged partner protein, as indicated below. In no case is the level of the AD- or DBD-fusion protein different whether the co-expressed partner interacts or not (see S5 Data). A. In lane 1, samples are from yeast co-expressing unlabeled DBD-Pum[RBD^+^], which comigrates with AD-Nos. B. Accumulation of AD-Nos RBD is indistinguishable in yeast co-expressing DBD-fusions to Pum[NTR + RBD] and Pum[NTR^+^]. C and D. Samples are from yeast in which the unlabeled DBD-fusions indicated below are co-expressed. Underlying data are in S5 Data.
(TIF)

S3 Fig Expression levels of the DBD-and AD-fusion proteins used in the two-hybrid experiments of Fig 7. Similar to S2 Fig, panels A-D show the relative levels (in bold below) of the RBD and [NTR + RBD] fusion proteins to either Hs Pum1 or Hs Pum2, as indicated above. Each sample is from yeast in which the relevant partner is not HA-tagged and therefore not detected. In A-C, the level of the AD- or DBD-fusion protein being measured is not significantly different whether the co-expressed partner interacts or not (see S5 Data). In D, there is a 2-fold difference in the level of both DBD-RBD and DBD-[NTR + RBD] upon co-expression of AD-RBD (vs. AD-[NTR + RBD]); however, the relative level of the two DBD-fusions is the same in these cases. We do not understand the reason that fusions bearing the Pum1 or Pum2 NTRs migrate as a cluster of bands. Underlying data are in S7 Data.
(TIF)

S4 Fig Characterization of RNA-binding of Pum fragments bearing partial deletions of the NTR. A. Schematic drawing to scale showing the two deletion derivatives used in the yeast three-hybrid experiments here and in Fig 8. The drawing also shows the structure of Pum[RBD*], which is expressed in transgenic flies. B. Relative expression in yeast of three Pum fragments is indicated in bold below. One reason we used Pum[NTR(Δ2) + RBD] for the experiments of Fig 8 is that it is expressed at a relatively high level, for unknown reasons. C. Three-hybrid experiments in which binding of the two NTR deletion derivatives is compared with binding of Pum[NTR + RBD], using the wt NRE and a panel of mutant NREs, similar to the experiment of Fig 2C. Underlying data are in S8 Data.
(TIF)

S1 Data Information used to generate Fig 1D. ΔΔG values are from S2 Table in [19] and β-galactosidase measurements from S7 Data.
(XLSX)

S2 Data Underlying data for the experiments of Fig 2.
In addition to data summarized in Fig 2, the file contains data from the following controls. An experiment showing that neither (1) the RNA encoded by the RPR1 vector only nor (2) any of the NREs used in Fig 2C stimulate the LacZ reporter appreciably in the absence an AD-fusion to one of the Pum fragments are in the tab named “NRE auto-activation controls.” An experiment showing no appreciable stimulation of the LacZ reporter by any of the three AD-fusions to Pum fragments in yeast expressing the RPR1 empty vector RNA are in the tab named “empty RNA vector controls.” The tab named “AD deletion controls” shows that, for all the proteins used in the three- and four-hybrid experiments reported here, stimulation of the LacZ reporter in each case is dependent on the vector-encoded GAL4 AD. To perform these control experiments, for each AD-fusion used in this report, we prepared a plasmid encoding an otherwise identical fusion bearing an in-frame deletion that removes the entire GAL4 AD, leaving the SV40 NLS intact. We were particularly concerned that cryptic signals in the Pum or Nos NTRs, which contain large disordered regions, might fortuitously activate transcription. If so, β-galactosidase levels would reflect not only recruitment to the LacZ reporter but also the sum of transcriptional activation by GAL4 and the cryptic signal. As shown in the file, only the Hs Pum1 and Pum2 N-terminal extensions appear to harbor such cryptic signals and so fusions bearing them were not used in this work. We showed that stimulation by the HsPum2 cryptic signal in three-hybrid experiments depends on RNA-binding; although the signal robustly stimulates transcription in the absence of the GAL4 AD when co-expressed with the NRE, there is no stimulation when co-expressed with a site to which Pum does not bind, the Smaug Response Element (SRE).
(XLSX)

S3 Data Underlying data for the experiments of Figs 3 and S1.
In addition to the underlying β-galactosidase measurements for Fig 3C and quantitation of the Western blots for Fig 3D, the file contains the following information. One tab in the file shows auto-activation controls for the four-hybrid experiments of Fig 3C, showing no significant binding of AD-Nos in yeast expressing (1) various NLS-Puf domain fusions and the RPR1 vector-encoded RNA only (i.e., bearing neither NRE nor FBE) (2) protein encoded by the empty NLS-fusion vector (i.e., neither Pum nor FBF2) and either the NRE- or FBE-containing hybrid RNAs. Another tab shows no substantial difference between the level of RNA-binding activity of Pum[NTR + RBD] and its F1367S mutant derivative in three-hybrid experiments. Additional, tabs contain the source data for S1B-S1D Fig.
(XLSX)

S4 Data Underlying data for the experiments of Fig 4.
The file contains underlying β-galactosidase measurements for Fig 4A as well as NRE auto-activation controls, showing that (1) none of the NREs used in these experiments stimulates the LacZ reporter when co-expressed with NLS-Pum[NTR + RBD] but in the absence of AD-Nos and (2) AD-Nos does not bind to any of the NREs in NLS empty vector control cultures that express no Pum protein. The file also contains the scoring of segmentation for Fig 4B as well as details of the RT-qPCR measurements of transgenic expression of the NRE 2A and NRE 2C mRNAs. Another tab in the file contains underlying β-galactosidase measurements for Fig 4C.
(XLSX)

S5 Data Underlying data for the experiments of Figs 5 and S2.
(XLSX)

S6 Data Underlying data for the experiments of Fig 6. The file also contains controls showing that (1) none of the NREs used in these experiments stimulates the LacZ reporter when co-expressed with NLS-Pum[NTR + RBD] in the absence of AD-Nos and (2) AD-Nos does not bind to any of the NREs in NLS empty vector control yeast that express no Pum protein.
(XLSX)

S7 Data Underlying data for the experiments of Figs 7 and S3.
The file also has a tab showing no binding of any of the four Hs Pum fusions to the RPR1 RNA encoded by the empty vector. Another tab shows that in control experiments, none of the three human Nos proteins binds to a wt NRE on its own (e.g., in the absence of a NLS-Puf domain fusion). Another tab shows that none of the NLS-fusions to human Pum fragments tested recruits any of the three human Nos proteins to a wt NRE in four-hybrid experiments.
(XLSX)

S8 Data Underlying data for the experiments of Figs 8 and S4.
The file also contains empty vector AD and RNA controls, which show that none of the RNAs in Fig 8A auto-activates the LacZ reporter and that none of the [NTR + RBD] fusions binds to the vector-encoded RPR1 RNA.
(XLSX)

S9 Data Plasmids used in this work.
(XLSX)

S1 Text A discussion of additional controls for the experiments of Figs 2-4.
(PDF)
