# Supplementary figures and images for: Significant roles in RNA-binding for the amino-terminal regions of Drosophila Pumilio and Nanos
Source: PLoS Genet. 2025 Mar 31;21(3):e1011616. doi: 10.1371/journal.pgen.1011616 (PMC11981137; doi:10.1371/journal.pgen.1011616)

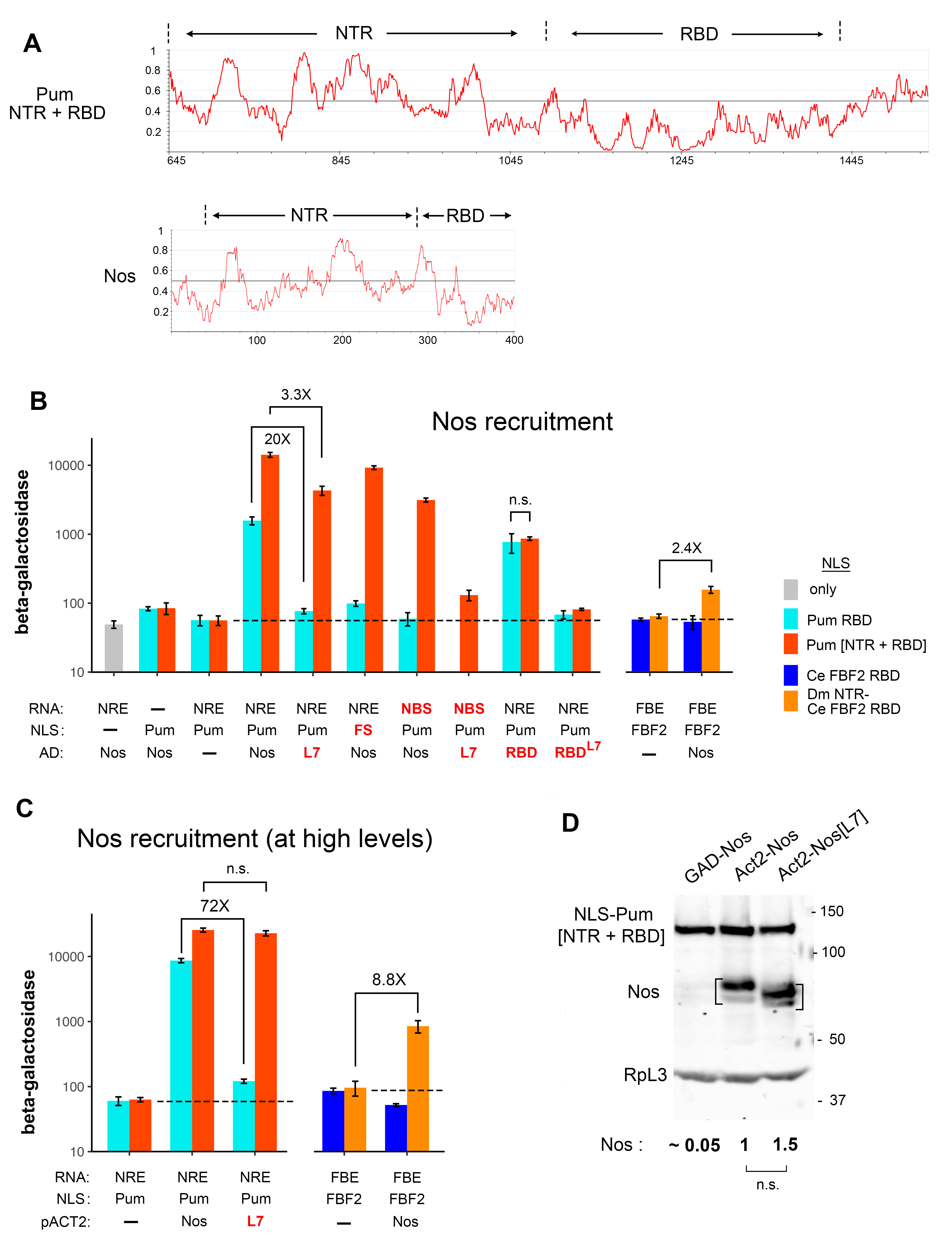

Supplement: S1 Fig — A. Plots of the probability of disorder calculated using IUPred2A [62] (y-axis) for Pum[NTR + RBD] and Nos, plotted versus residue number of the full-length proteins on the x-axis. B. Similar to the experiment of Fig 3C, but comparing Nos recruitment by Pum[NTR + RBD] and by Pum RBD. Note that the level of NLS-Pum[NTR + RBD] is higher than the level of NLS-Pum RBD (Fig 3D); this may contribute somewhat to the higher level of LacZ reporter activity for the former protein, although we argue in the text that the level of AD-Nos (rather than the level of NLS-Pum) is the primary limiting factor in these experiments. Experiments with the FBE are the same as in Fig 3C, shown for comparison to the results of over-expressing AD-Nos in C, as follows. C. A subset of the experiments in B, but showing recruitment of Nos expressed from pACT2 plasmid derivatives that direct the expression of higher levels of protein, as shown in D. D. Relative Nos expression levels (in bold below), reveal that Nos is expressed to an approximately 20-fold higher level in the experiments of C compared with the experiments of B (or Fig 3C). Note that AD-Nos and NLS-Pum[NTR + RBD] are approximately equimolar (S3 Data). There is no significant difference in accumulation of wt Nos and NosL7, consistent with the observation that the mutant protein is stable in embryos [63]. Underlying data in S3 Data. (TIF) [file pgen.1011616.s001.tif]

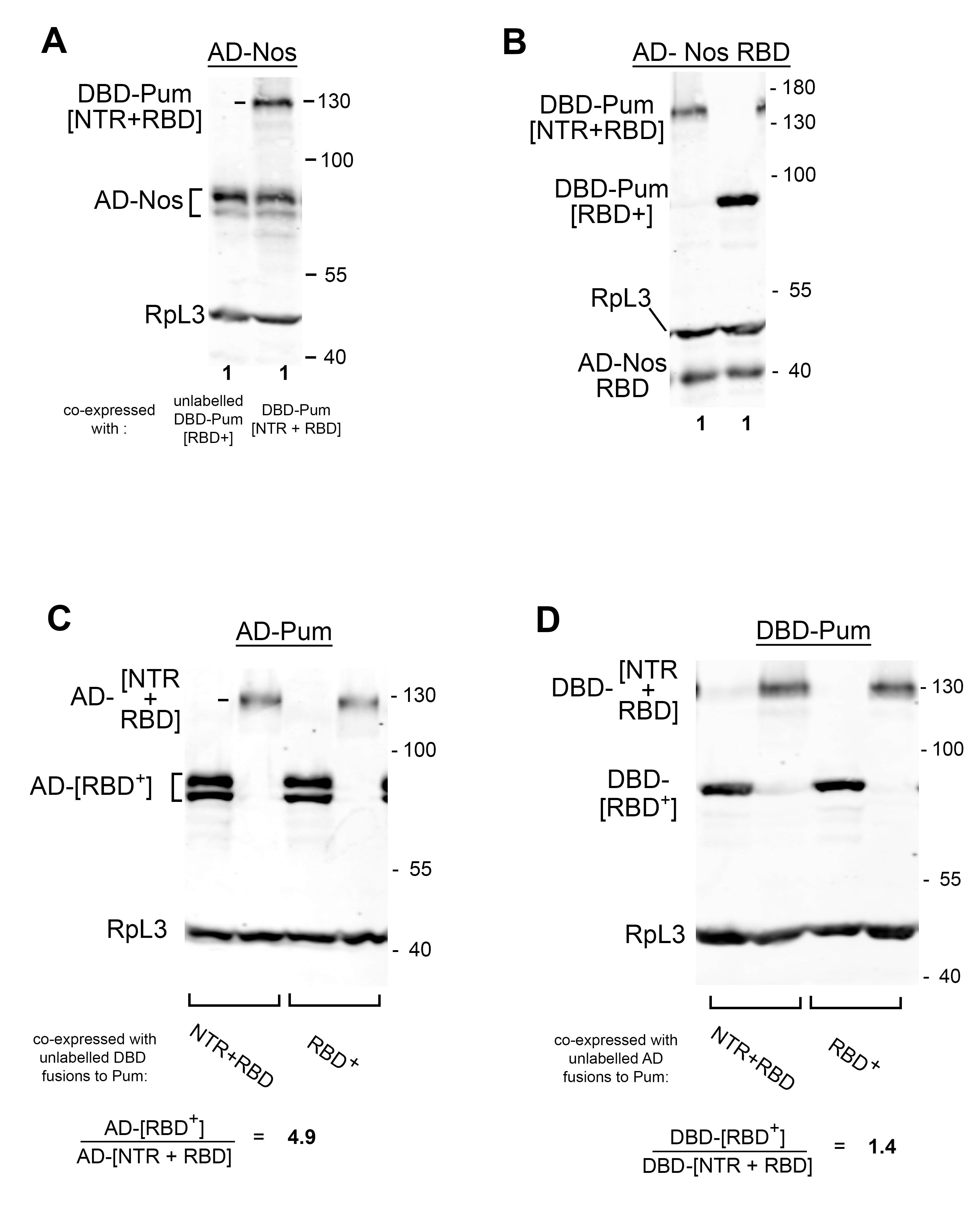

Supplement: S2 Fig — Each panel is a Western blot to measure the relative levels of proteins named above the image and quantitated below in bold. All samples are from yeast co-expressing the relevant factors shown in the two-hybrid experiments of Fig 5. In A, C, and D, where AD- and DBD-fusion proteins comigrate, yeast co-expressed an otherwise identical but untagged partner protein, as indicated below. In no case is the level of the AD- or DBD-fusion protein different whether the co-expressed partner interacts or not (see S5 Data). A. In lane 1, samples are from yeast co-expressing unlabeled DBD-Pum[RBD+], which comigrates with AD-Nos. B. Accumulation of AD-Nos RBD is indistinguishable in yeast co-expressing DBD-fusions to Pum[NTR + RBD] and Pum[NTR+]. C and D. Samples are from yeast in which the unlabeled DBD-fusions indicated below are co-expressed. Underlying data are in S5 Data. (TIF) [file pgen.1011616.s002.tif]

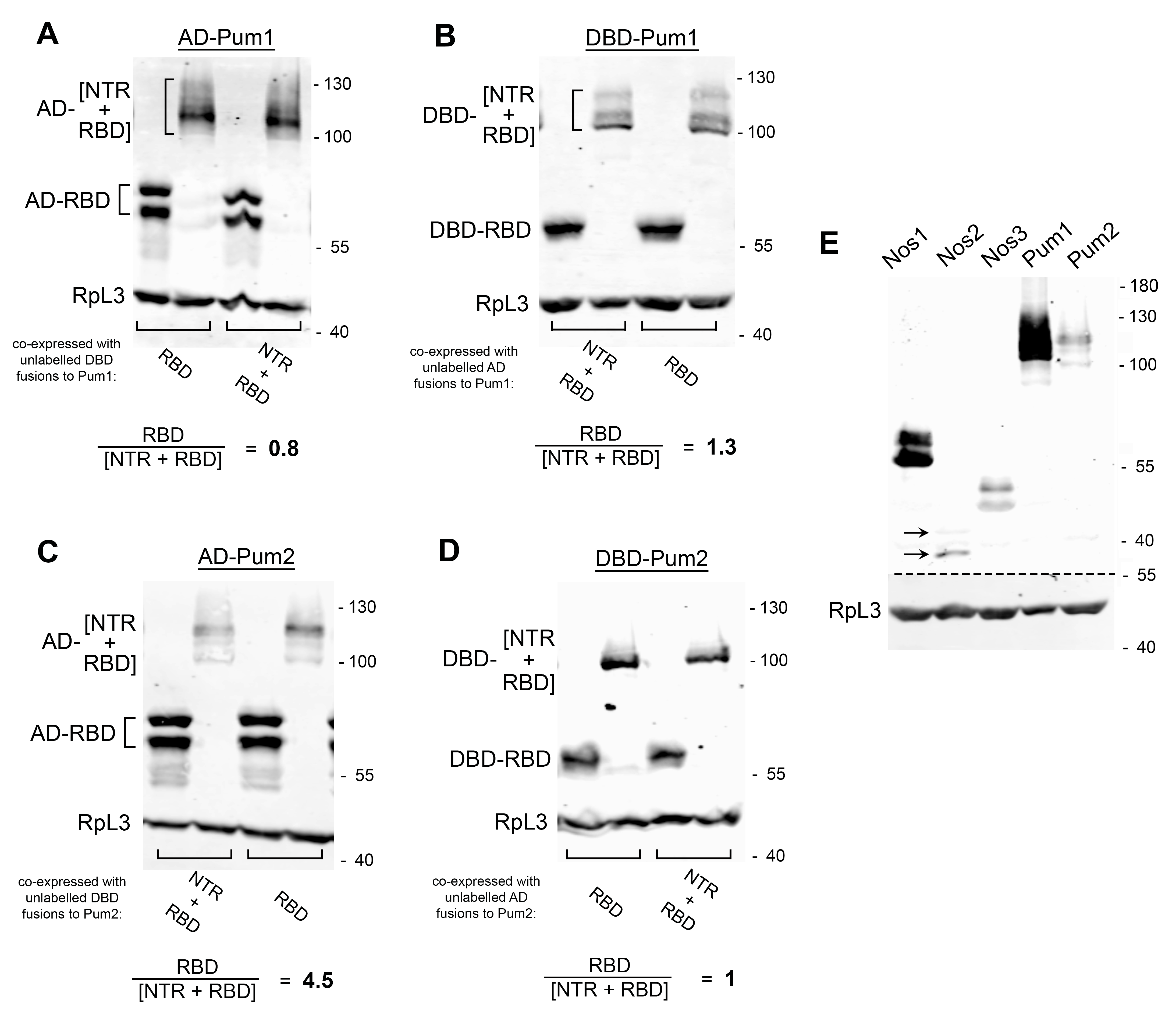

Supplement: S3 Fig — Similar to S2 Fig, panels A-D show the relative levels (in bold below) of the RBD and [NTR + RBD] fusion proteins to either Hs Pum1 or Hs Pum2, as indicated above. Each sample is from yeast in which the relevant partner is not HA-tagged and therefore not detected. In A-C, the level of the AD- or DBD-fusion protein being measured is not significantly different whether the co-expressed partner interacts or not (see S5 Data). In D, there is a 2-fold difference in the level of both DBD-RBD and DBD-[NTR + RBD] upon co-expression of AD-RBD (vs. AD-[NTR + RBD]); however, the relative level of the two DBD-fusions is the same in these cases. We do not understand the reason that fusions bearing the Pum1 or Pum2 NTRs migrate as a cluster of bands. Underlying data are in S7 Data. (TIF) [file pgen.1011616.s003.tif]

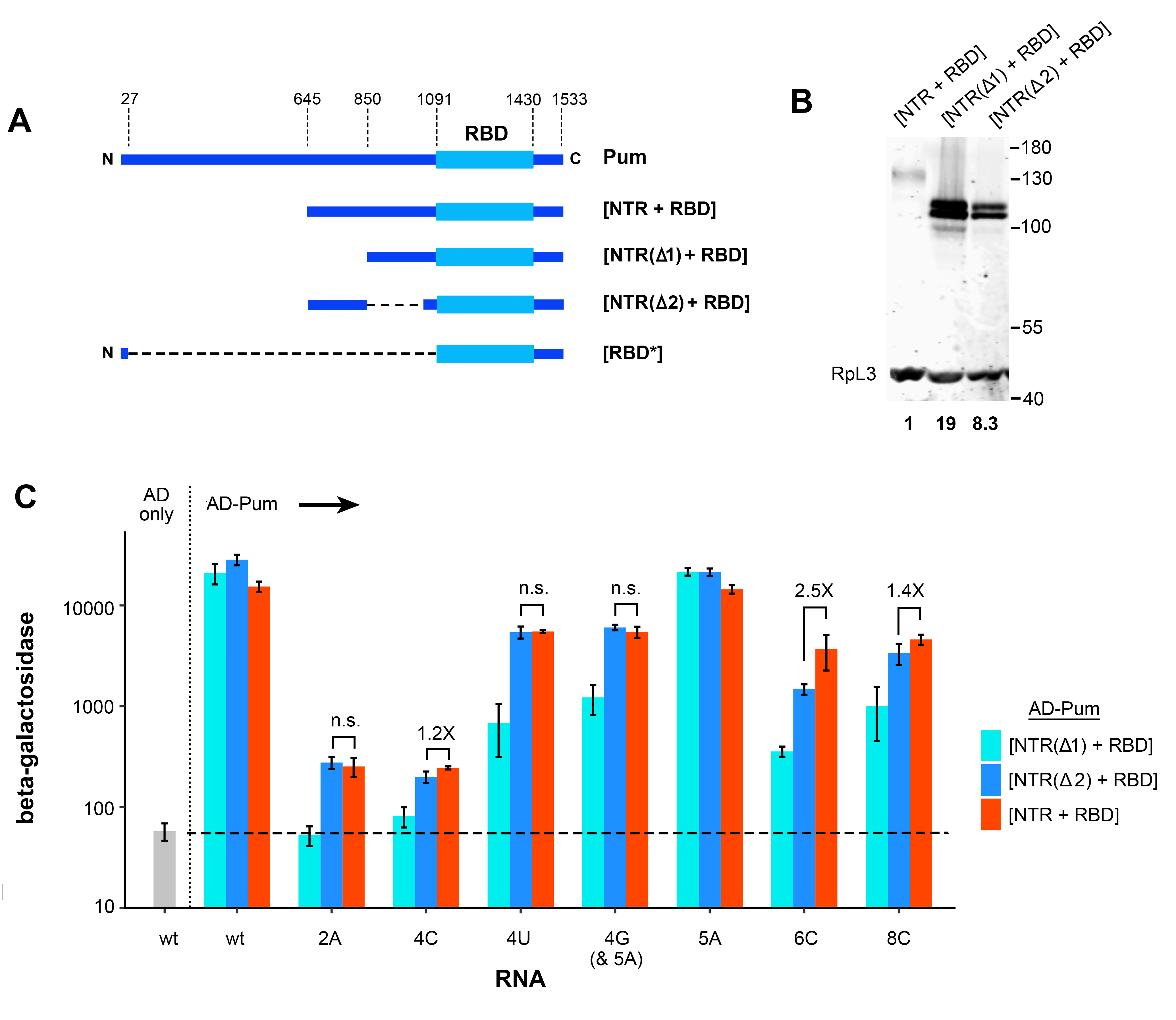

Supplement: S4 Fig — A. Schematic drawing to scale showing the two deletion derivatives used in the yeast three-hybrid experiments here and in Fig 8. The drawing also shows the structure of Pum[RBD*], which is expressed in transgenic flies. B. Relative expression in yeast of three Pum fragments is indicated in bold below. One reason we used Pum[NTR(Δ2) + RBD] for the experiments of Fig 8 is that it is expressed at a relatively high level, for unknown reasons. C. Three-hybrid experiments in which binding of the two NTR deletion derivatives is compared with binding of Pum[NTR + RBD], using the wt NRE and a panel of mutant NREs, similar to the experiment of Fig 2C. Underlying data are in S8 Data. (TIF) [file pgen.1011616.s004.tif]
