# Supplementary material for: Significant roles in RNA-binding for the amino-terminal regions of Drosophila Pumilio and Nanos
Source: PLoS Genet. 2025 Mar 31;21(3):e1011616. doi: 10.1371/journal.pgen.1011616 (PMC11981137; doi:10.1371/journal.pgen.1011616)
Supplement: S1 Text — (PDF) [file pgen.1011616.s014.pdf]

S1 Text

Three-hybrid specificity controls.

In addition to the controls shown in Fig 2, we have performed a number of other control experiments that support the ideas that (1) the beta-galactosidase values measured reflect specific binding and (2) the ability of each fusion to activate transcription is entirely dependent on the vector-encoded GAL4 AD. As a result, it is valid to compare binding of different proteins, provided the relative level of each is taken into account.

The background level of β-galactosidase in yeast containing the AD only empty vector control is not substantially greater for any of the mutant NREs than for the wt NRE, which is shown in grey on the left in Fig 2C (see S2 Data). Thus, none of the RNA "baits" in our three hybrid experiments significantly auto-activates transcription of the reporter gene.

We ruled out the possibility that any of the proteins assayed in this work bears a fortuitous transcriptional activation signal that might augment activity of the vector-encoded GAL4 AD common to them all. For each AD-Puf domain fusion assayed in this report, we prepared a derivative bearing an in-frame deletion of the GAL4 AD and asked whether the resulting ΔAD protein-fusion activates transcription when co-expressed with the appropriate, cognate RNA-binding site. As shown in S2 Data, none of the ΔAD protein-fusions used in this work activates the three-hybrid reporter substantially above background levels, supporting the conclusion that β-galactosidase levels faithfully report site occupancy and not differential activation of the LacZ reporter.

The level of Nos is limiting in the experiments of Fig 3.

Several lines of evidence show that, when expressed from derivatives of the pGAD vector, the level of Nos is not saturating in our four-hybrid experiments.

First, the NLS-RBD Pum fusion is expressed at a 15-fold lower level than is NLS-Pum[RBD$^+$]. But Pum RBD recruits Nos to only a 1.8-fold lower level, and thus the level of Nos appears to be the main factor limiting ternary complex formation (S4 Data).

Second, we expressed higher levels of the AD-Nos fusion to test whether these would yield higher levels of ternary Nos/Pum/NRE complex or allow "inappropriate" Nos recruitment in the absence of the interactions between the Nos and Pum RBDs that govern regulation in embryos. The level of AD-Nos in the experiments described above is near the threshold of detection, and thus cannot be measured reliably. However, we can over-produce AD-Nos an estimated 20-fold using the pACT2 vector (S1 Fig D). At this higher level, the Pum RBD recruits Nos to a greater extent, stimulating β-galactosidase production 144-fold as compared with only 28-fold at the lower level of AD-Nos (S1 Fig and S3 Data). Nos recruitment, which in this case is mediated solely by interactions between the RBDs and with the NRE, is still specific, by the criterion that recruitment of Nos^{L7} is reduced to near-background levels (S1 Fig C). But apparently the higher level of AD-Nos in yeast with the pACT2 vector drives inappropriate complex formation by Pum[NTR + RBD], which no longer discriminates between Nos and Nos^{L7} (S1 Fig C).

Third, at the higher level of Nos, the Dm-Ce chimera recruits Nos to the FBE, stimulating β-galactosidase production 8.8-fold above the basal level (in the absence of any known interaction between the Drosophila Nos and C. elegans FBF2 RBDs) (S1 Fig C).

Identical mRNA levels of the 2C and 2A *hb* reporter mRNAs.

The regulatory system that governs abdominal segmentation in vivo is finely poised, such that relatively small changes in the level of maternal *hb* mRNA substrate can alter the number of abdominal segments that develop [40]. To be certain that the apparent difference between repression activity mediated by the 2C and 2A mutant NREs is not due to a difference in the level of transgene-encoded mRNA, we measured transgenic mRNA by RT-qPCR in 0-1 hour old embryos. The 2C and 2A transgenic mRNAs are expressed at the same level (S4 Data).
